# Retrospective study of late radiation-induced damages after focal radiotherapy for childhood brain tumors

**DOI:** 10.1371/journal.pone.0247748

**Published:** 2021-02-26

**Authors:** Claudia Cavatorta, Silvia Meroni, Eros Montin, Maria C. Oprandi, Emilia Pecori, Mara Lecchi, Barbara Diletto, Ombretta Alessandro, Denis Peruzzo, Veronica Biassoni, Elisabetta Schiavello, Marco Bologna, Maura Massimino, Geraldina Poggi, Luca Mainardi, Filippo Arrigoni, Filippo Spreafico, Paolo Verderio, Emanuele Pignoli, Lorenza Gandola

**Affiliations:** 1 Medical Physics Unit, Fondazione IRCCS Istituto Nazionale dei Tumori, Milano, Italy; 2 Department of Electronics Information and Bioengineering (DEIB), Politecnico di Milano, Milan, Italy; 3 Neuro-oncological and Neuropsychological Rehabilitation Unit, Scientific Institute IRCCS E. Medea, Bosisio Parini, Lecco, Italy; 4 Pediatric Radiotherapy Unit, Fondazione IRCCS Istituto Nazionale dei Tumori, Milano, Italy; 5 Bioinformatics and Biostatistics Unit, Fondazione IRCCS Istituto Nazionale dei Tumori, Milano, Italy; 6 Neuroimaging Lab, Scientific Institute IRCCS E. Medea, Bosisio Parini, Lecco, Italy; 7 Pediatric Oncology Unit, Fondazione IRCCS Istituto Nazionale dei Tumori, Milano, Italy; Nathan S Kline Institute, UNITED STATES

## Abstract

**Purpose:**

To study a robust and reproducible procedure to investigate a relation between focal brain radiotherapy (RT) low doses, neurocognitive impairment and late White Matter and Gray Matter alterations, as shown by Diffusion Tensor Imaging (DTI), in children.

**Methods and materials:**

Forty-five patients (23 males and 22 females, median age at RT 6.2 years, median age at evaluations 11.1 years) who had received focal RT for brain tumors were recruited for DTI exams and neurocognitive tests. Patients’ brains were parceled in 116 regions of interest (ROIs) using an available segmented atlas. After the development of an ad hoc, home-made, multimodal and highly deformable registration framework, we collected mean RT doses and DTI metrics values for each ROI. The pattern of association between cognitive scores or domains and dose or DTI values was assessed in each ROI through both considering and excluding ROIs with mean doses higher than 75% of the prescription. Subsequently, a preliminary threshold value of dose discriminating patients with and without neurocognitive impairment was selected for the most relevant associations.

**Results:**

The workflow allowed us to identify 10 ROIs where RT dose and DTI metrics were significantly associated with cognitive tests results (p<0.05). In 5/10 ROIs, RT dose and cognitive tests were associated with p<0.01 and preliminary RT threshold dose values, implying a possible cognitive or neuropsychological damage, were calculated. The analysis of domains showed that the most involved one was the “school-related activities”.

**Conclusion:**

This analysis, despite being conducted on a retrospective cohort of children, shows that the identification of critical brain structures and respective radiation dose thresholds is achievable by combining, with appropriate methodological tools, the large amount of data arising from different sources. This supported the design of a prospective study to gain stronger evidence.

## Introduction

Late neurocognitive sequelae in childhood brain tumor survivors have been extensively documented [1–4] and are known to correlate to several risk factors, such as radiotherapy (RT) doses, fraction size, target volume and young patient age [2, 5–7]. RT is crucial to achieve cure in the majority of pediatric brain tumors, but due to its intrinsic neurotoxicity, it is also one of the main causes of iatrogenic sequelae, together with tumor effects, surgery and chemotherapy. Late brain injuries become manifest as progressive neurologic impairment, affecting cognitive abilities and neuropsychological domains (i.e. IQ scores, attention, working memory, processing speed etc.) with a consequent increasing need of rehabilitation interventions. Furthermore, neuropsychological effects may include social, emotional and behavioral disorders [8], leading to a significantly decreased quality of life, compared with peers [1, 6, 9, 10]. RT brain damage usually involves white matter injuries and demyelination (or structural degradation) of axon fibers, causing the disruption of trans-synaptic communications [11]. These tissue alterations cannot be effectively assessed with morphological Magnetic Resonance Imaging (MRI). Many papers showed that Diffusion Tensor Imaging (DTI) is a reliable tool for evaluating white matter (WM) and grey matter (GM) changes [12–14]. DTI can assess alterations in morphologically normal appearing areas and can be used as early indicator of post treatment neurotoxicity [10, 15–22]. An increasing number of studies have been investigating the correlation between RT doses and DTI values in specific brain areas for both whole brain and focal RT [7, 16, 17, 21, 23–27], while other studies found associations between neurocognitive outcomes and radiation dose [28–30]. Finding any correlation between RT dose, neurocognitive measurements and GM-WM indicators of “integrity” (like DTI-derived variables) may help to expand our knowledge about the local, long-term cerebral structural and cognitive effects also of low radiation doses and may be used to optimize treatment planning procedures with dose constraints for each eloquent brain functional area. Many efforts have been made so far to obtain dose-effect curves for different areas of child brain at different ages; specific areas, such as the frontal and temporoparietal lobes, hippocampus and other supratentorial structures, seem to play an important role in the genesis of cognitive decline [3, 6, 23, 31, 32]. Despite all efforts, robust experimental dose-effect curves for specific pediatric brain areas have not been defined yet.

In this scenario, this study was performed to explore a reliable procedure to define possible correlations between neurocognitive outcome after focal RT for childhood brain cancer and low RT doses, WM and GM alterations detected by DTI. Special interest was taken in brain areas receiving low radiation doses, since these areas are far from target structures that have been affected by surgery and by the tumor itself. The final goal was to define a workflow that could allow the identification of brain regions, receiving low RT dose levels, significantly correlating with cognitive impairment and structural alterations as measured by DTI, as prerequisites for future studies aimed at refining treatment planning criteria for focal RT to improve patients’ outcomes and quality of life.

## Methods and materials

From 2014 to 2017 we enrolled 45 children with malignant brain tumor, whose oncologic therapy included focal RT and at least 3 years of follow-up in complete remission of their tumours (patients treated from 2002 to 2013). Table 1 reports the main clinico-pathological characteristics of the enrolled children. Institutional Review Board of Fondazione IRCCS Istituto Nazionale dei Tumori approved the study and written consent was obtained from all participants and their legal guardians.

**Table 1:** Clinical overview of the enrolled patients.

	N	%
**Total patients**	45	
**Sex**		
Male	23	51.1
Female	22	48.9
**Tumor site**
Supratentorial	29	64.4
Infratentorial	16	35.6
**Histological Diagnosis**		
Ependymoma	16	35.6
Medulloblastoma (MBL)	2	4.4
Atypical teratoid rhabdoid tumor (AT/RT)	2	4.4
Diffuse Intrinsic Pontine Glioma (DIPG)	3	6.7
Supratentorial pleomorphic sarcoma	1	2.2
High-Grade Glioma (HGG)	7	15.6
Supratentorial Primitive Neuroectodermal Tumors (S-PNET)	5	11.1
Germinoma	8	17.8
Adamantinomatous Craniopharyngioma (ACP)	1	2.2
**Curative surgery**		
Yes	35	77.8
No	10	22.2
**Treatment**		
No chemotherapy	6	13.3
Systemic chemotherapy	26	57.8
Systemic chemotherapy+myeloablative Thiotepa	13	28.9
**Prescribed Dose (Gy)**		
30.6	4	8.9
36	4	8.9
54	23	51.1
59.4	14	31.1
**Age** (years)	Median (range)	
Age at RT	6.2 (1.1–22.5)	
Age at test evaluation	11.1 (4.4–25.9)	

Clinical characteristics of the enrolled patients.

Original RT treatment plans and related Computed Tomography images (planning-CT) were retrospectively retrieved and re-calculated to standardize calculation algorithm, dose-grid and data format. Forty-three patients received 3D Conformal RT, with an average number of 5 fields per patient, and 2 were treated with Volumetric Modulated Arc Therapy (VMAT). For each patient we selected a post-operative MRI (defined as MR0) usually contemporary to the start of RT. All the MR0 exams included T1-weighted sequences acquired with a 1.5 Tesla scanner. All patients or their legal guardians signed an informed consent to participate in the study.

### Cognitive and neuropsychological assessment and MRI acquisition

At study enrollment (i.e. at least 3 years after RT) all children received a cognitive and neuropsychological assessment using standardized tests normalized by age and provided with well-defined thresholds indicating impairment. Concurrently, patients underwent a 3 Tesla follow-up brain MRI exam. A description of all tests is reported in Table 2. The references of the tests administered to patients are reported in S1 Table. Patients were assessed with a different number of tests, according to their age.

**Table 2:** Overview of the administered cognitive and neuropsychological tests.

			**FSIQ**			
***Cognitive Assessment***	**Test**	**Age**	**VIQ**	**PIQ**	**Indexes**	**Tot.**
						**Subtest**
	**Wechsler Preschool and Primary Scale of Intelligence(WPPSI III)**	2.5 ≤ years < 4	• Information	• Block Design	-	5
			• Receptive Vocabulary	• Block Assembly		
			• (Pictures naming)			
		4 ≤ years < 7.5	• Information	• Block Design	• Processing Speed**	14
			• Vocabulary	• Matrix Reasoning		
			• Word Reasoning	• Picture Concepts		
			• (Comprehension)	• (Picture completion)		
			• (Similarities)	• (Block Assembly)		
			• Receptive Vocabulary	• Coding**		
			• Pictures naming	• Symbol Search**		
	**Wechsler Intelligence Scale for Children(WISC III)**	6 ≤ years < 17	• Information	• Picture Completion	• Freedom from Distractibility*	13
			• Similarities	• Coding**		
			• Arithmetic*	• Picture Arrangement	• Processing speed**	
			• Vocabulary	• Block Design		
			• Comprehension	• Object Assembly		
			• (Digit Span)*	• (Symbol search)**		
				• (Mazes)		
	**Wechsler Adult Intelligence Scale(WAIS-R)**	≥17 years	• Information	• Block Design	-	11
			• Similarities	• Object Assembly		
			• Vocabulary	• Picture Completion		
			• Arithmetic	• Picture Arrangement		
			• Digit Span	• Digit Symbol		
			• Comprehension			
			**General Quotient**			
	**Griffiths Mental Development Scales (GMDS)**	**0-2 years**	• Locomotor			5
			• Personal-Social			
			• Hearing and Language			
			• Eye and Hand Co-ordination			
			• Performance			
		**3-8 years**	• Locomotor			6
			• Personal-Social			
			• Language			
			• Eye and Hand Co-ordination			
			• Performance			
			• Practical Reasoning			
***Neuropsychological Assessment***		**Test**		**Age**	**Index**	**N. of Index**
	**Memory**	Rey Complex Figure		≥4 years	• Recall	1
	**Attention**	Conners Kiddie Continuous Performance Test (K-CPT)		4 ≤ years < 7 years	• Reaction Times	3
					• Omissions	
		Continuous Performance Test (CPT)		≥6 years	• Commissions	
	**Executive Functions**	Modified Card Sorting Test (MCST)		4 ≤ years ≤13 years	• Category	8
					• Tot. Correct Answers	
		Wisconsin Card Sorting Test (WCST)		≥6 years	• Tot. Errors	
					• Ambiguous Answers	
					• Perseverative Answers	
					• Perseverative Errors	
					• Non-perseverative Errors	
					• Conceptual Answers	
	**Praxic Abilities**	Perdue Pegboard (PP)		≥5 years	• Dominant Hand	4
					• Non-dominant Hand	
					• Both-hands	
					• Assembly	
		Rey Complex Figure		≥4 years	• Copy	1

Overview of the cognitive and neuropsychological tests administered to the patients and/or their parents. The number of sub-evaluations of each test is reported and, where applicable, aggregated indexes calculated from the sub-evaluations scores are shown. Test between brackets are additional. Subtests indicated with (*) contribute to the calculation of Index Freedom from Distractibility, while those indicated with (**) contribute to the calculation of Index Processing Speed.

Cognitive and neuropsychological subtests were analyzed individually and also grouped per domain as shown in S2 Table. The domain’s evaluation was considered impaired if a patient had shown impairment for at least 33% of the subtests belonging to the domain.

Baseline evaluations performed at the time of RT were available for 18/45 patients. A description of the available baseline evaluations is reported in S3 Table and S1 File.

The details of the 3 Tesla MRI examination protocol performed at the time of enrollment are reported in S4 Table. The acquisition parameters were chosen in order to provide a good trade-off among accuracy, reliability and angular resolution. DTI processing was performed using TORTOISE software (NIH Pediatric Neuroimaging Diffusion Tensor MRI Center, Bethesda, MD, USA). The preprocessing pipeline included a motion correction step, a correction of image distortions (e.g. eddy current, magnetic field susceptibility, etc.) using the non-distorted T2-weighted volume as reference [33, 34], a realignment to the AC-PC plane and an up-sampling to a final voxel resolution of 1.5x1.5x1.5 mm^3^. Data were visually inspected to detect remaining artifacts and/or wrong preprocessing results. Corrupted volumes were discarded from the subsequent analysis. The DTI tensor was computed using the non-linear least square method described by Chang et al. [35] and Fractional anisotropy (FA), Mean Diffusivity (MD), Axial Diffusivity (AD) and Radial Diffusivity (RD) maps were calculated for each subject.

### Data processing

All images used in this study were processed and transported into the same frame of reference by using a homemade multistep registration framework *ad hoc* developed for this project [36] adopting widely used, cross-platforms, open-source image registration toolkits (ITK) [37] and openMP®. The registration process had to consider possible disease and treatment-related anatomical changes over time and the physiologic anatomical growth of the child from the time of RT to DTI evaluation, during years of follow-up. Anatomical changes were then modeled using a combination of rigid and non-rigid registrations. Firstly, planning-CT and dose distribution were registered on MR0. Secondly, the follow-up MRI/DTI acquired many years later was registered to MR0 so that all images lay in the same reference coordinate space. The registration between CT and MR0 was performed using only rotations and translation, since both exams refer to the same time point and complex morphological changes of the head are not expected. The registration between the follow-up MRI/DTI and the MR0 was performed using both affine (translation, rotation, scaling and shear) and non-rigid transformations (B-spline), as detailed in [36]. B-spline non-rigid transformation was necessary to account for the substantial anatomical changes related to the normal children’s growth. The cost function for both types of registration (rigid and non-rigid) was a combination of mutual information and normalized gradient field as detailed in [36].

The patients’ brain parcellation was performed using the brain atlas included in the Automatic Anatomical Labeling (AAL) software [38]. The current version of the atlas provides the segmentation of cortical, sub-cortical and cerebellar structures identified with the acronyms described in S5 Table. The brain atlas and its co-registered T1 MRI were registered to the follow-up MRI/DTI.

The quality of each step of the image registration process was visually inspected by experienced radiation oncologists on 3D Slicer software, after accurate contouring of selected anatomical structures in axial, sagittal and coronal views. The selected anatomical structures were brain Organs At Risk (OARs) usually contoured for RT dose distribution planning and other structures important for the quality assessment of rotations: skull, falx, eyes, brainstem, nose, tentorium. At the end of the process, we obtained 116 regions of interest (ROIs) (see S5 Table) of cortical, sub-cortical and cerebellar structures, each characterized by a mean RT dose value and mean MD, AD, RD and FA values. To highlight the role of lower RT doses, a special focus was then dedicated to ROIs characterized by mean dose lower than 75% of the RT dose prescription. The 75% isodose was chosen as in our dataset it was shown to include target regions more likely to have been affected by surgery, tumor itself and higher radiation doses.

### Statistical analysis

The statistical analysis was carried out according to the following steps:

- use of Kruskal Wallis test to study the association between clinical–demographic variables (sex, age, tumor histology, surgery, chemotherapy, prescription dose, tumor site, age at evaluations, time-lapse between RT and evaluations) and the percentage of non impaired test normalized according to the average of the overall number of the performed tests;
- evaluation of the relationship between AD, RD, MD and FA obtained in the 116 ROIs using the Spearman correlation coefficient (ρ_s_);
- evaluation of the relationship between mean RT dose values in the 116 ROIs and corresponding mean DTI-metrics values using ρ_s_;
- assessment of the associations between cognitive and neuropsychological performances and RT doses or DTI values separately in each ROI by means of the Kruskal-Wallis test. The Kruskal-Wallis test results were focused according to the inferred assumption on the direction of the relationships between the dose values and the remaining variables: negative for FA values and test results and positive for AD, RD and MD values [13]. Firstly (first analysis) all ROIs, irrespective to the level of dose, were considered for the analysis. The neurocognitive scores of each patient were categorized as falling below average (impaired) or within average outcome (non impaired), according to the standardized threshold for each specific test. To overcome computational limitations and to guarantee the patients representativeness, only cognitive evaluations with a minimum number (n = 5) of both impaired and non-impaired outcomes were considered. Differences in dose, AD, RD, MD and FA values distributions between patients with impaired or non-impaired performances (in a given neurocognitive test) were considered statistically significant at p<0.05. Then the associations found between tests scores and RT doses or DTI values were compared to identify shared ROIs for which both dose and DTI values were associated with patients impairment within a subtest. An additional analysis (second analysis) was then performed excluding the ROIs with mean doses higher than 75% of the dose prescription in order to consider only low dose areas far from the RT target regions. The list of the excluded ROIs is shown in S6 Table. Moreover, in such analysis, the neurocognitive baseline tests results (available for 18/45 patients) were considered: patients that already showed an impairment at baseline assessment were excluded from the analysis of the specific test, as the impairment was likely caused by other factors. Therefore, only associations detected in the first analysis that remained significant according to the second analysis were considered in order to obtain more robust results.
- generation of Receiver Operating Characteristic (ROC) curves focusing on the strongest associations with respect to RT dose obtained through the Kruskal-Wallis test (i.e. with p<0.01) that remained significant according to the second analysis. ROC curves allowed the evaluation of a predictive value of low RT dose on cognitive test scores in terms of AUC (Area Under the Curve) as well as to obtain a preliminary optimal dose cut-point (maximization of Youden’s index) able to explain the difference between impaired and non impaired patients referred to ROIs involved in the most significant associations. Due to the exploratory nature of this study and coherently with its generating hypothesis purpose, adjustments for multiple testing were not performed in order not to fail to highlight any important finding [39].
- use of Fisher’s exact test to investigate the relations between clinical-demographic categorical variables and impaired/non impaired status of the tests involved in the most robust associations found in the low dose investigation.

The second analysis was finally repeated considering the neurocognitive test grouped per domain. Statistical analysis was performed with SAS software 9.4 (SAS Institute Inc., Cary, NC, USA).

## Results

Overall, 44/45 patients were the object of this analysis (one patient was excluded due to an unreliable image registration result caused by the presence of a post-operative pneumocephalus in the MR0 images).

**Fig 1:**
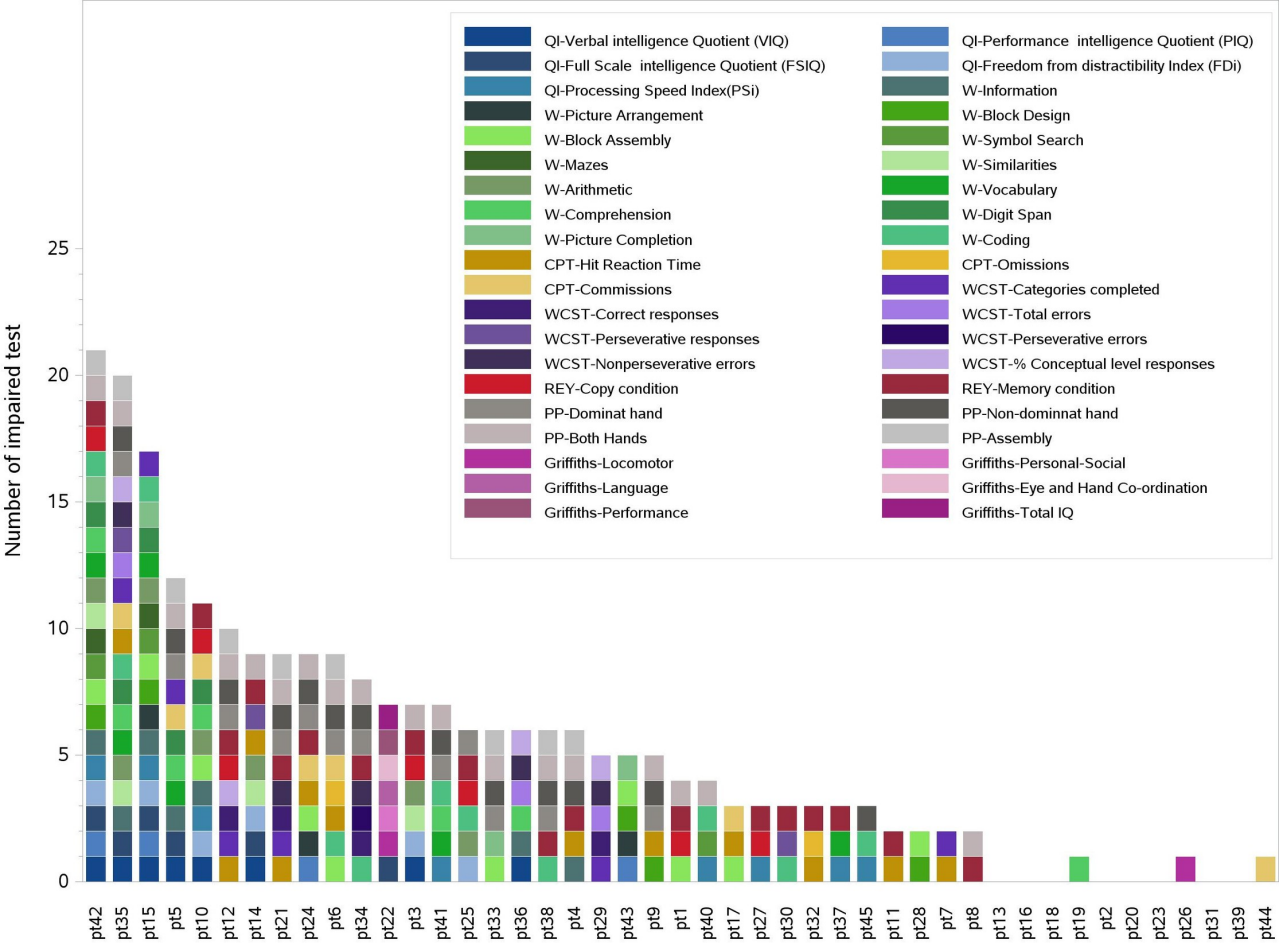
Overview of the subtests indicating impairment of each patient. Colored blocks refer to subtests scores under the impairment thresholds. Eight patients presented no impairment.

Considering the whole set of administered tests, 8/44 patients had only non-impaired results and 3/44 patients showed only one sub-evaluation score under the threshold value. The 8 non impaired patients did not share common characteristics (sex, age, tumor histology, surgery, chemotherapy, prescription dose, tumor site, age at evaluations, time-lapse between RT and evaluations). The 3 patients with only one sub-evaluation indicating impairment were about 2 years old at the time of RT and their tumors were located in the IV ventricle. Two of them were classified as ependymoma at diagnosis and one as AT/RT, all underwent radical surgery and received same radiation doses. Despite these similarities, all of them presented different kinds of impaired sub-test evaluations. The maximum number of impaired sub-evaluations obtained by a single patient was 21/34. An overview of patients’ performances at the time of evaluations is shown in Fig 1 whereas the overview of patients’ neurocognitive performances with respect to the investigated clinical characteristics are shown in S1 Fig. No significant associations were highlighted by this analysis.

Since we required the presence of at least 5 patients each for both endpoints (impaired and non-impaired), only 22 cognitive and neuropsychological tests were considered for the analysis (see S7 Table).

Spearman’s Correlation coefficients evaluated in each ROI showed the presence, in most regions, of a positive association between AD, MD and RD values, and of a less relevant inverse association between FA and the other metrics. AD, MD and RD values resulted correlated with RT doses, reaching ρ_s_ values close to 0.80. Low correlations were observed between FA and doses with an extreme observed ρ_s_ of -0.58. The highest correlations between dose and DTI metrics were observed in Frontal_Mid_Orb_L_1, Frontal_Mid_Orb_R_1, Occipital_Mid_R, SupraMarginal_L and Vermis_9.

**Fig 2:**
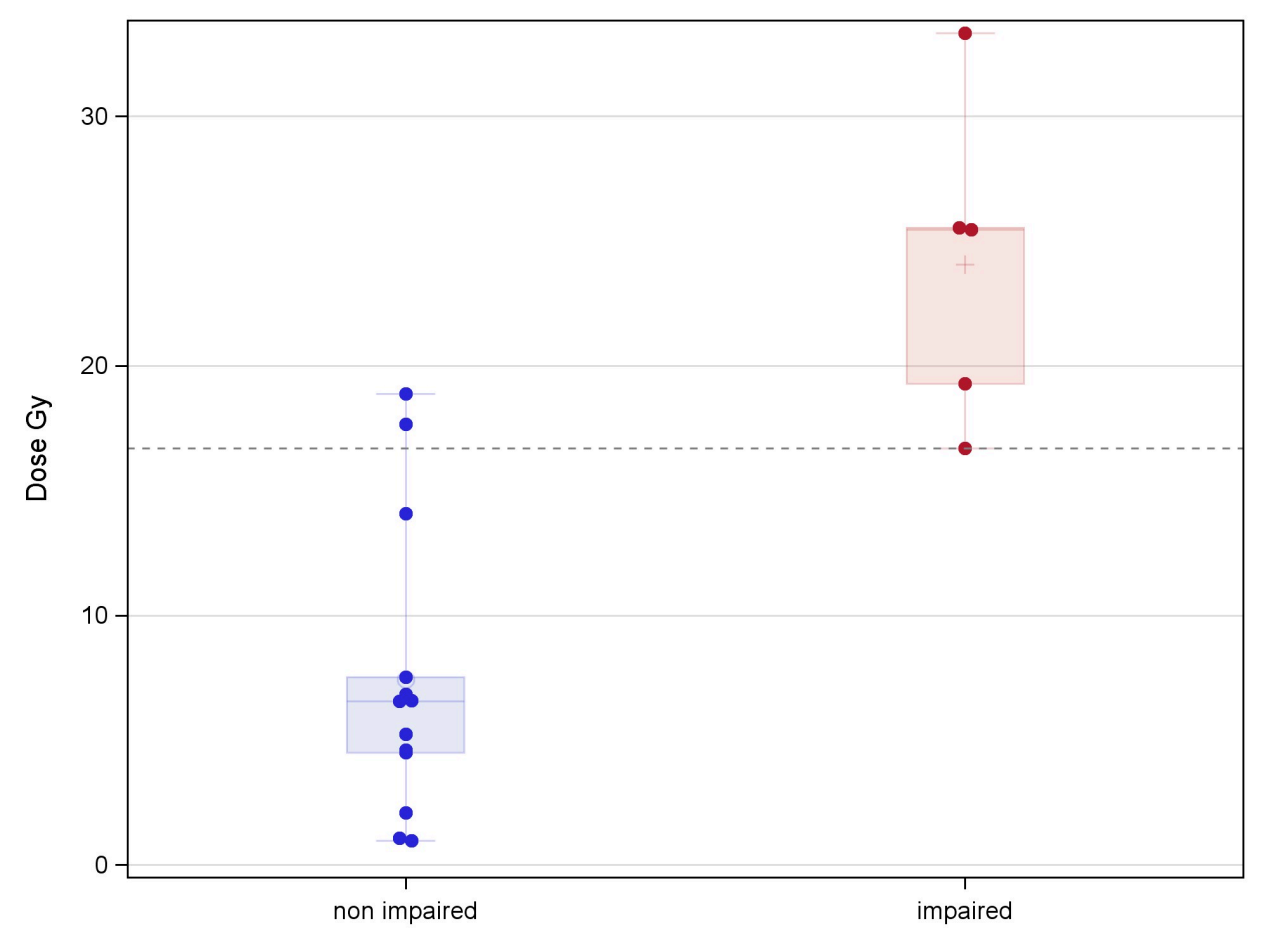
Example of the RT dose values distribution for impaired and non impaired patients for a test. Example of the statistically significant difference between the distributions of RT dose values for patients with non-impaired performances (N = 13) with respect to those with impaired performances (N = 5) in a specific cognitive evaluation (sub-evaluation Dominant hand of PP) and for a specific ROI (Vermis_4_5).

**Fig 3:**
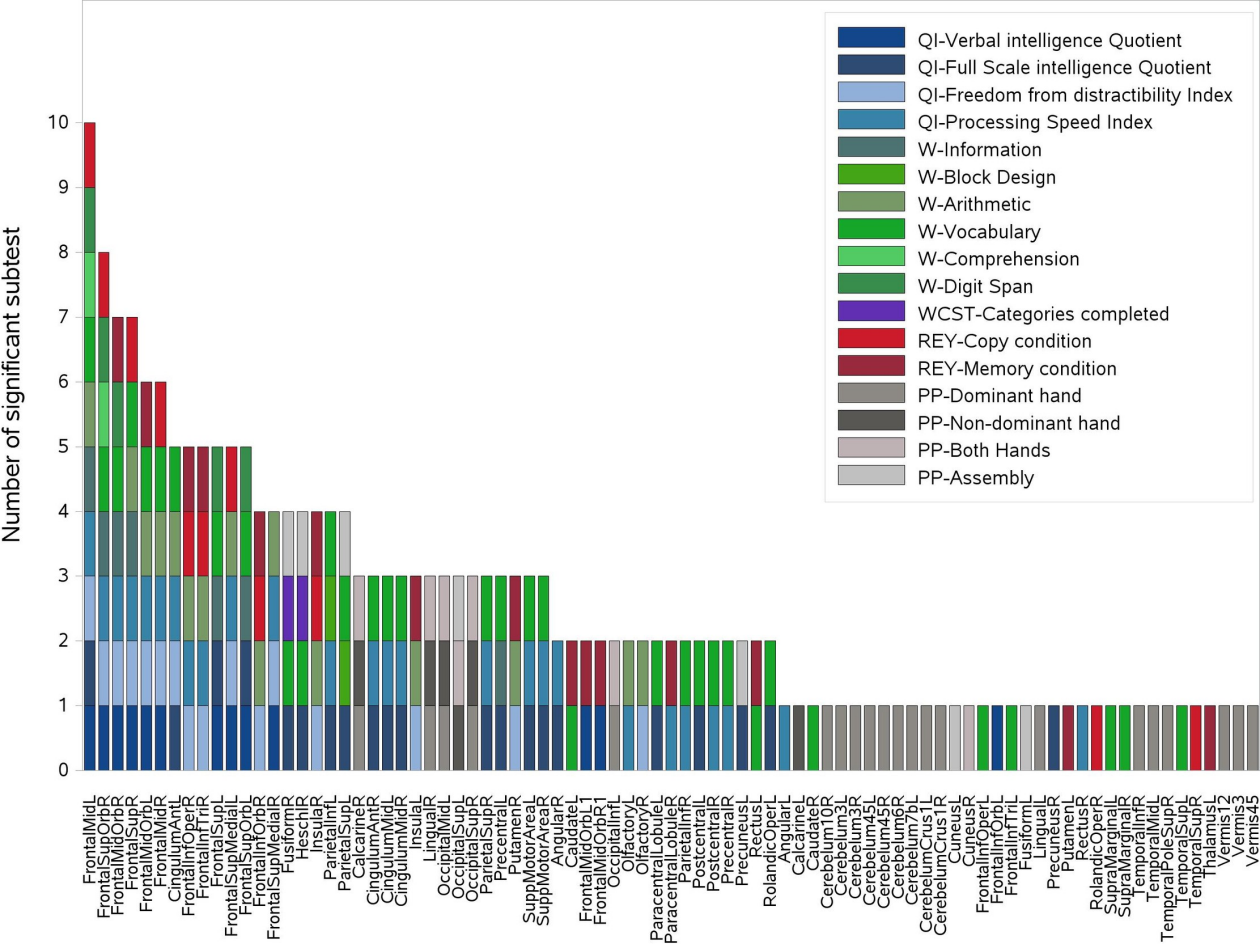
Representation of the 210 significant associations between dose and cognitive and neuropsychological evaluations scores. The associations were obtained through the Kruskal-Wallis analysis and 83 ROIs and 17 different tests were involved. For each ROI reported on the x-axis, all scores showing a significant association with dose are reported on the y-axis in the form of colored boxes. IQ: Intelligence quotient, W: WPPSI-WISC-WAIS tests, REY: Rey Complex Figure tests, WCST: Wisconsin Card Sorting Test, PP: Perdue Pegboard test.

Fig 2 shows examples of the statistically significant difference between the distributions of dose values in patients with non-impaired performances with respect to those with impaired performances in a specific neurocognitive evaluation. A total of 210 significant associations (p<0.05) were obtained through the Kruskal-Wallis dose analysis, involving 83 ROIs and 17 different neurocognitive sub-evaluations/indexes (Fig 3). Among the 17 neurocognitive scores resulting associated to the dose, the most frequent belonged to WISC/WAIS Test, IQ indexes and Purdue Pegboard Test.

A summary of the significant associations observed between DTI values and neurocognitive performances are shown in supplementary figures (S2–S5 Figs).

By focusing on the relationships involving a specific ROI and sub-evaluation/index and more than one independent variable, we found that 40 out of the 210 associations were statistically significant for both dose and MD (p<0.05), involving 27 ROIs and 11 different sub-evaluations/indexes (Table 3); 32 out of 40 were statistically significant also for both RD and AD (S6 Fig).

**Table 3:** Significant associations found for the first and the second analysis and the domain analysis.

First Analysis		Second Analysis				Domain analysis
ROI_name	subtest	ROI_name	subtest	AUC value and 95% CI	Dose cut-off (Gy)	Domain
**Cerebelum_10_R**	Dominant Hand (PP)	**Cerebelum_10_R**	Dominant Hand (PP)			
**Cerebelum_3_R**	Dominant Hand (PP)					
**Cerebelum_4_5_L**	Dominant Hand (PP)					
**Cerebelum_4_5_R**	Dominant Hand (PP)					
**Cerebelum_Crus1_L**	Dominant Hand (PP)	**Cerebelum_Crus1_L**	Dominant Hand (PP)			
**Cerebelum_Crus1_R**	Dominant Hand (PP)					
**Cingulum_Ant_L**	Freedom from distractibility index	**Cingulum_Ant_L**	Freedom from distractibility index	0.91 (0.78;1)	9.9	school-related abilities
	Full scale IQ					
**Frontal_Inf_Tri_L**	Vocabulary (WPPSI/WISC/WAIS)					
**Frontal_Mid_Orb_L**	Verbal IQ					
	Vocabulary (WPPSI/WISC/WAIS)					
**Frontal_Mid_R**	Freedom from distractibility index	**Frontal_Mid_R**	Freedom from distractibility index			
**Frontal_Sup_L**	Information (WPPSI/WISC/WAIS)					
	Digit span (WISC/WAIS)					
	Vocabulary (WPPSI/WISC/WAIS)					
**Frontal_Sup_Medial_L**	Verbal IQ					
**Frontal_Sup_R**	Arithmetic (WISC/WAIS)					
	Freedom from distractibility index					
	Information (WPPSI/WISC/WAIS)					
	Verbal IQ					
	Vocabulary (WPPSI/WISC/WAIS)					
**Heschl_R**	Assembly (PP)					
**Insula_L**	Freedom from distractibility index	**Insula_L**	Freedom from distractibility index	0.92(0.79;1)	12.9	school-related abilities
**Insula_R**	Arithmetic (WISC/WAIS)	**Insula_R**	Arithmetic (WISC/WAIS)	0.89(0.77;1)	18.2	school-related abilities/ memory
	Freedom from distractibility index		Freedom from distractibility index	0.91(0.78;1)	18.2	school-related abilities/ memory
**Occipital_Mid_L**	Dominant Hand (PP)	**Occipital_Mid_L**	Dominant Hand (PP)			
	Non Dominant Hand (PP)		Non Dominant Hand (PP)			
**Occipital_Sup_R**	Dominant Hand (PP)					
	Non Dominant Hand (PP)					
**Olfactory_L**	Processing speed index					
**Paracentral_Lobule_L**	Full scale IQ	**Paracentral_Lobule_L**	Full scale IQ	0.88(0.75;1)	7.7	
	Vocabulary (WPPSI/WISC/WAIS)		Vocabulary (WPPSI/WISC/WAIS)			
**Parietal_Inf_L**	Full scale IQ					
	Vocabulary (WPPSI/WISC/WAIS)					
**Parietal_Sup_L**	Full scale IQ					
**Postcentral_L**	Full scale IQ					
**Temporal_Mid_L**	Dominant Hand (PP)	**Temporal_Mid_L**	Dominant Hand (PP)			
**Temporal_Pole_Sup_R**	Dominant Hand (PP)					
**Vermis_3**	Dominant Hand (PP)					
**Vermis_4_5**	Dominant Hand (PP)	**Vermis_4_5**	Dominant Hand (PP)	0.97(0.90;1)	16.7	

List of the ROIs in which we found a significant association (i.e. p <0.05) between cognitive and neuropsychological evaluation score and both dose values and MD values. On the left the results of the first analysis which considered all the ROIs, while on the right are showed the results of the second analysis performed excluding ROIs with mean doses higher than 75% of the prescription. Preliminary cut-off dose values and AUC values were obtained in the analysis performed for the ROIs having an association with dose with p<0.01. The third column highlights the associations significant also for the domain analysis.

The second analysis performed excluding the ROIs with mean doses higher than 75% of the dose prescription and taking into account patients’ baseline impairment, confirmed 13 out of the 40 significant associations observed in the first analysis. Among these 13 associations, 6 showed a p<0.01 for dose. In details, 5 ROIs were involved and 4 different sub-evaluations/indexes belonging to Purdue Pegboard Test, QI indexes and WISC/WAIS Test. These 6 associations also showed AUC values ranging between 0.88 and 0.97. Cut-off dose values above which patients were more likely to experience a specific cognitive impairment are reported for these 6 associations (Table 3). The preliminary threshold dose values ranged from 7 Gy (Paracentral_Lobule_L) to 18Gy (Insula_R). The neurocognitive performances involved in the above 6 significant associations were analyzed with respect to the patients clinical characteristics. The only significant association found was related to the Dominant hand subtest (PP) with respect to the clinical variable “age at RT”. In particular patients 5–10 years old showed no impairment in the Dominant hand subtest in contrast to the other classes (Fisher’s exact test: p = 0.003 see S7 Fig).

As concerns the domain analysis, S8 Fig reports the prevalence of impairment in each involved subtest. By pursuing the analysis we observed that, among the 5 ROIs for which we computed a cutoff value, Cingulum Ant L, Insula R and Insula L were involved also in the results of the domain analysis (see Table 3 –last column). For all of these three ROIs the impairment of school-related abilities resulted associated with both RT dose and MD values (p value <0.05).

## Discussion

Therapeutic irradiation of the brain in children with brain tumors poses major issues, due to the known risk of severe neurocognitive toxicity. Nevertheless, RT remains an irreplaceable local therapy for many tumors. The more recent 3D conformal techniques adopting a large number of fields improved distribution of high doses around the target as compared to 2D techniques but increased the volume of normal tissues absorbing lower doses. This aspect is even more pronounced with the newer intensity modulated RT techniques such as VMAT or Tomotherapy. While the volume receiving the RT high-dose levels is smaller than with 3D conformal techniques, the overall irradiated volume is usually larger. The effect of low radiation doses on different healthy brain structures has not yet been clearly defined but the increasing use of all IMRT techniques requires an effort to study this issue. Connor et al. [15] in a recent work concluded that even low doses to WM may not completely prevent microstructural damage visible on DTI images, even though it is not clear whether and to what extent microstructural damage translates into a clinically significant effect. Hypothesizing that each anatomical structure has different radiosensitivity, it would be useful to steer low doses towards less sensitive areas, to allow for better sparing of more susceptible structures thus preserving specific functions. Yet, for most child brain structures a tolerance dose for radiation has not been determined. In this work we present a workflow designed to identify brain structures that may be more susceptible to radiation effects including low dose effects and to obtain threshold doses for these regions.

Since no baseline measures of DTI metrics were available for this study, we considered the cognitive test score as the major index of neurological impairment, as it has a standardized threshold. Starting from a large amount of data arising from different sources, the application of appropriate methodological tools allowed us to identify the ROIs and sub-evaluations/indexes that resulted significantly associated to one or more of the five variables of interest (dose, FA, AD, MD and RD). Some associations lost significance in the second analysis when the dose cut-off of 75% of the dose prescription was considered to exclude ROIs including tumor bed areas. A possible explanation is that these associations were probably due to other clinical factors such as surgery or disease itself as well as high therapeutic doses delivery. Still present in the second analysis was the anterior portion of the paracentral lobule, that was found to be associated to Vocabulary (VOC) and Full Scale IQ, thus suggesting a higher sensitivity to low RT doses. These associations can be explained considering that this region is part of the frontal lobe. Yokota et al. [40] reported that brain regions that positively correlated with FSIQ included the paracentral lobule, whose anterior part (the supplementary motor area) is also involved in the motor aspects of speech production [41].

Significant associations between cerebellar dose and poor scores in the Purdue Pegboard test, which evaluates fine motor dexterity, were found both in the first and in the second analysis. As shown in Table 3, in the second analysis that excluded ROIs involved by tumor, surgery and therapeutic RT doses, the number of cerebellar ROIs associated with impaired tests was reduced. The remaining associations likely highlight ROIs with higher sensitivity to low RT doses. It is well known that the cerebellum controls the timing and pattern of muscle activation during movement. The cerebellar vermis receives information from the spinal cord about the sense of touch and proprioception, contains representations of the body and helps control the execution of movements. Associations in cerebellar ROIs between DTI metrics values and test results, mostly related to cognitive and neuropsychological domain, were also found but, due to the lack of basal DTI values, further investigations are needed before drawing conclusions. These associations are interesting, since increasing evidence in the literature suggests a major role for the cerebellum in complex cognitive operations [32, 42].

Significant associations with PP scores were also found for Occipital Mid L and Temporal Mid L doses. Indeed the first area has an important role in coding object weight prior to grasping [43] and in object-directed action [44]. The second area is involved in many functions among which, motion [45] and grasp observation [46] and probably also in self-grasp observation, by a mechanism of mirroring. Also the temporal sulcus is involved in these activities [47].

Notably, the associations of temporal lobes and hippocampi with cognitive tests and dose values found in this work were not highly significant, as opposed to several paper in literature suggesting significant associations between increasing dose to these structures and decline in neurocognitive skills [3, 23, 28, 31, 48, 49]. However, Acharya et al. [28], in a recent paper regarding 80 children with low grade glioma, found a significant association between hippocampi doses above 40 Gy and memory decline. This result can contribute to explain our finding considering that in our series two-thirds of the patients received doses lower than 40 Gy to the hippocampi. Nevertheless, these preliminary results will have to be confirmed by further prospective studies.

Freedom from Distractibility (FDI) was found to be associated with the anterior Cingulum, which has been linked to executive attention and cognitive control [50]. Our results are in accordance with Connor et al. [25] who reported that the Cingulum bundle was among the most dose-sensitive regions that showed variations in DTI metrics. Also our finding of the link between FDI and the Insula is reasonable, because recent researches on the Default Mode Network (DMN) demonstrated the role of this structure in state-to-state switching: the Insula allows DMN disengagement and foster the engagement of specific brain network necessary for the task during rest-to-task transitions [51].

After the first and the second analysis our study allowed us to identify low dose thresholds for some specific ROIs. Indeed, for the most significant associations between test scores and ROI dose values (p<0.01), the dose-effect aspect was further investigated, identifying preliminary dose threshold levels between 7 and 18 Gy (Table 3).

Comparing the single test analysis and the domain analysis, it turns out that some ROIs are shared. More precisely the Insula and the Cingulum Ant are both associated with the Freedom from Distractibility (single test analysis) and the school related abilities (domain analysis). This seems reasonable, for the motivations reported above about role of the Insula in the DMN [51] and the role of the Cingulm Ant in executive attention and cognitive control [50]. Indeed, the Freedom from Distractibility Index is a measure of attention, concentration and working memory, that are skills and functions required in the academic tasks.

Moreover, the Insula is also related to the memory domain. Several studies suggest that the Insula is involved in numerous functions, namely auditory and salience processing and attention orientation. Therefore, the Insula has the role to integrate external sensory stimuli with internal signals, to manage interactions and switches between the DMN, as mention above and central executive network, relevant for the maintenance and manipulation of information [52]. All these steps and elements play a significant role in the memory processes and learning.

The analysis of the domains, allowed us to confirm some of the most relevant associations within ROIs we identified through our subtest-specific analysis, but we think that the domain analysis could introduce variability for results comparison between different studies, especially concerning patient impairment definition because of lack of standardization. Objective tests are gold standard in measuring cognitive function, as suggested by the International Cognition and Cancer Task Force [53], because they have a well-defined cut-off and they are standardized by patient age. Therefore, we would prefer to focus future analysis on objective tests.

Our study provided a snapshot of the cognitive performances and DTI description of a population of children focally irradiated for brain tumor with 3 to 9 years of follow-up after irradiation. Due to the cross-sectional retrospective characteristics of the study, where a single evaluation after RT was available for the majority of children, we were unable to precisely distinguish the specific role of irradiation in inducing possible alterations from the tumor itself, previous surgery and/or chemotherapy or other confounding factors. An effort to clean data as much as possible was made by performing the second analysis, that helped us to remove associations that might be due to factors other than low radiation doses. Seventeen patients had known clinical risk factors for cognitive decline at the time of RT (hydrocephalus, frontal subdural fluid leaks, wide resections) [54] and therefore could represent a confounding factor concerning radiotherapy effects. For 9 of these patients we had baseline data available while 2 had no impaired tests at follow up. Thus, 6 out of 44 patients are at greater risk of introducing a bias in our association analysis. The lack of baseline evaluation, except for 18 patients, represents a noteworthy limit of the present study. Nevertheless literature supports the results obtained in this study and they could be considered reliable. Therefore, this work allowed us to generate preliminary results and to define a detailed workflow to be used in a prospective study designed for the investigation of low doses effects on children cognitive and neuropsychological performance after focal brain irradiation.

We can conclude that the main aim of the project, i.e. to identify critical brain structures where low dose values are associated with cognitive deficits and DTI values, is achievable, supporting the development of a prospective study (ongoing). The prospective study will allow us to better identify cognitive impairments mainly related to low RT doses only taking into account pre-existing damages and individual variations pointed out by basal and follow-up evaluations. Freedom from confounding factors will increase accuracy and reliability of cut-off dose levels, highlighting areas that are likely to play a crucial role in the emergence of late cognitive problems. The availability of cut-off dose levels will give the chance to optimize treatment planning procedures and improve the quality of life of children.

## Supporting information

S1 Fig Distribution of the neurocognitive performance of the patients with respect to the investigated clinical characteristics. The clinical characteristics considered are: gender, prescription dose, curative surgery, chemotherapy, thiotepa Y/N, tumor site, tumor site (supra/infratentorial), age at RT, time between RT and DTI. Colored blocks refer to subtests scores under the impairment thresholds. The list of subtests is shown in the upper left section. CT = chemotherapy, HR-MBL = High Risk Medulloblastoma, HD-TT = High Dose Thiotepa, HGG = High Grade Glioma, AT/RT = Atypical Teratoid Rabdoid Tumor, Nimo = Nimotuzumab, Vino = Vinorelbine, yrs = years.
(TIFF)

S2 Fig Representation of the 36 significant associations between FA and evaluations scores. The associations were obtained through the Kruskal Wallis analysis, involving 28 ROIs and 16 different tests. For each ROI reported on the x-axis, all scores showing a significant association with FA are reported on the y-axis in the form of colored boxes.
(TIF)

S3 Fig Representation of the 151 significant associations between AD and evaluations scores. The associations were obtained through the Kruskal Wallis analysis, involving 68 ROIs and 16 different tests. For each ROI reported on the x-axis, all scores showing a significant association with AD are reported on the y-axis in the form of colored boxes.
(TIF)

S4 Fig Representation of the 143 significant associations between RD and evaluations scores. The associations were obtained through the Kruskal Wallis analysis, involving 66 ROIs and 19 different tests. For each ROI reported on the x-axis, all scores showing a significant association with RD are reported on the y-axis in the form of colored boxes.
(TIF)

S5 Fig Representation of the 147 significant associations between MD and evaluations scores. The associations were obtained through the Kruskal Wallis analysis, involving 64 ROIs and 19 different tests. For each ROI reported on the x-axis, all scores showing a significant association with MD are reported on the y-axis in the form of colored boxes.
(TIF)

S6 Fig Representation of the 32 significant shared associations for MD, RD and AD. The associations involve 25 ROIs and 10 different tests. For each ROI reported on the x-axis, all scores showing a significant association with dose, MD, RD and AD, are reported on the y-axis in the form of colored boxes.
(TIF)

S7 Fig Bar-chart showing the percentage of patients with an impaired or non impaired subtest Dominant Hand of PP test, in the 4 groups, based on different age RT. (TIF)

S8 Fig Bar-chart showing the percentage of patients with an impairment (red) or not (blue) for each subtest grouped by domains. d1: General intellectual abilities, d2: Verbal abilities, d3: School-related abilities, d4: Memory, d5: Executive Functions, d6: Visuo-spatial and visuo-motor abilities.
(TIF)

S1 Table Cognitive, neuropsychological assessment references. (PDF)

S2 Table Cognitive and neuropsychological subtests grouped per domain. (PDF)

S3 Table Overview of the baseline evaluations. Note: x: impaired score; -: not applicable for patient age or not administered; FDI: Freedom from Distractability Index; FSIQ: Full Scale Intelligent Quotient; GMDS: Griffiths Mental Development Scales; K/CPT: Kiddie and Continuous Performance Test; M/WCST: Modified and Wisconsin Card Sorting Test; PIQ: Performance IQ; PP: Purdeue Pegboard; PSI: Processing Speed Index; REY: Rey Complex Figure; VIQ: Verbal IQ; W/GMDS: Wechsler and Griffiths Mental Development Scales; WISC: Wechsler Intelligence Scale for Children 3rd Edition; WPPSI: Wechsler Preschool and Primary cale of Intelligence, 3rd Edition.
(PDF)

S4 Table Overview of the MRI sequences acquired. (PDF)

S5 Table Anatomical description of the brain atlas ROIs acronyms. Abbreviations: ROI, regions of interest. From Tzourio-Mazoyer et al. [39].
(PDF)

S6 Table Number of the ROIs excluded with the second analysis out of the 44 used in the first analysis. (PDF)

S7 Table Subtests & indexes with at least 5 patients impaired and 5 non impaired. (PDF)

S1 File Overview of the baseline neurocognitive evaluation results. (PDF)
