# Supplementary figures and images for: Retrospective study of late radiation-induced damages after focal radiotherapy for childhood brain tumors
Source: PLoS One. 2021 Feb 26;16(2):e0247748. doi: 10.1371/journal.pone.0247748 (PMC7909688; doi:10.1371/journal.pone.0247748)

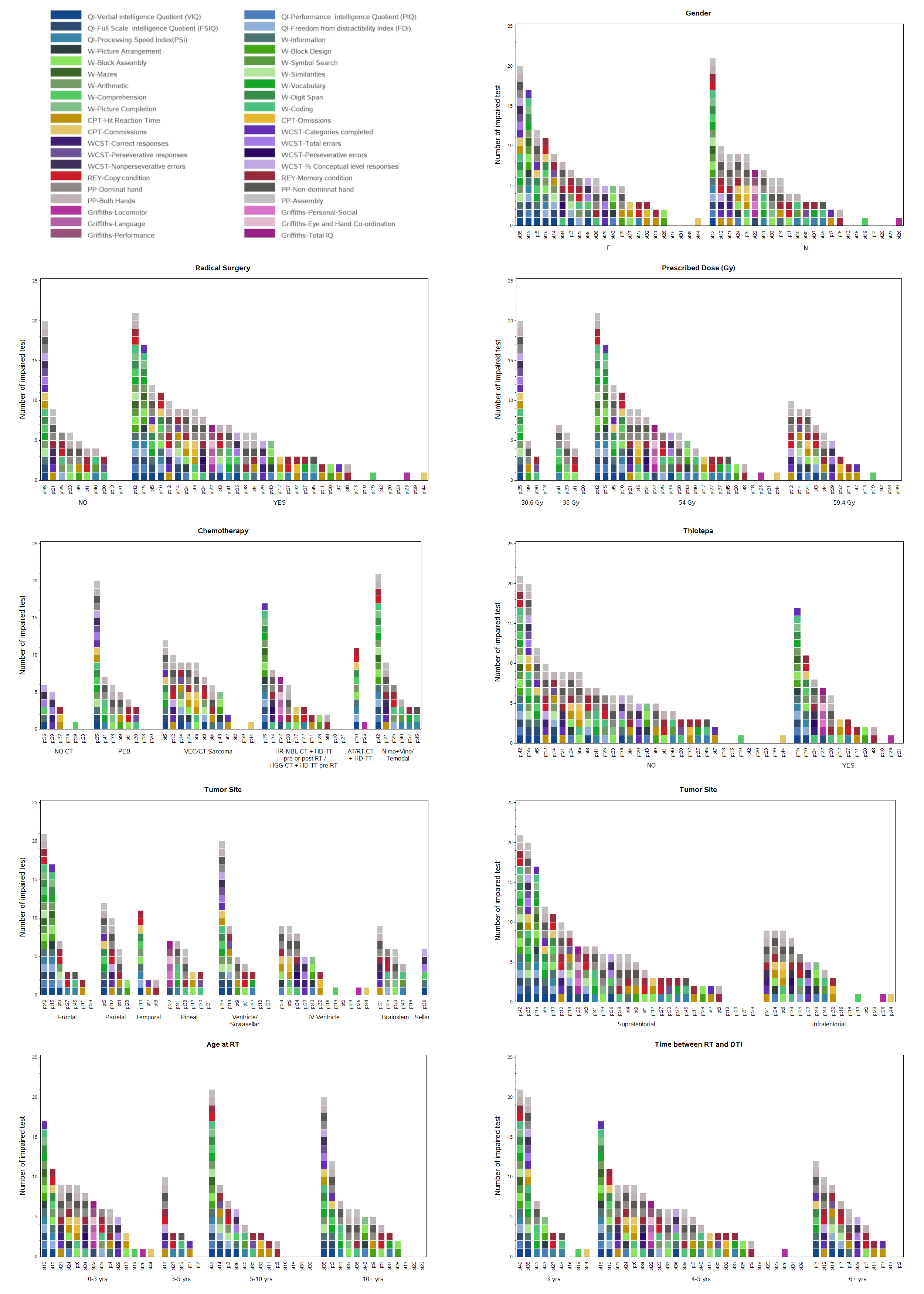

Supplement: S1 Fig — The clinical characteristics considered are: gender, prescription dose, curative surgery, chemotherapy, thiotepa Y/N, tumor site, tumor site (supra/infratentorial), age at RT, time between RT and DTI. Colored blocks refer to subtests scores under the impairment thresholds. The list of subtests is shown in the upper left section. CT = chemotherapy, HR-MBL = High Risk Medulloblastoma, HD-TT = High Dose Thiotepa, HGG = High Grade Glioma, AT/RT = Atypical Teratoid Rabdoid Tumor, Nimo = Nimotuzumab, Vino = Vinorelbine, yrs = years. (TIFF) [file pone.0247748.s001.tiff]

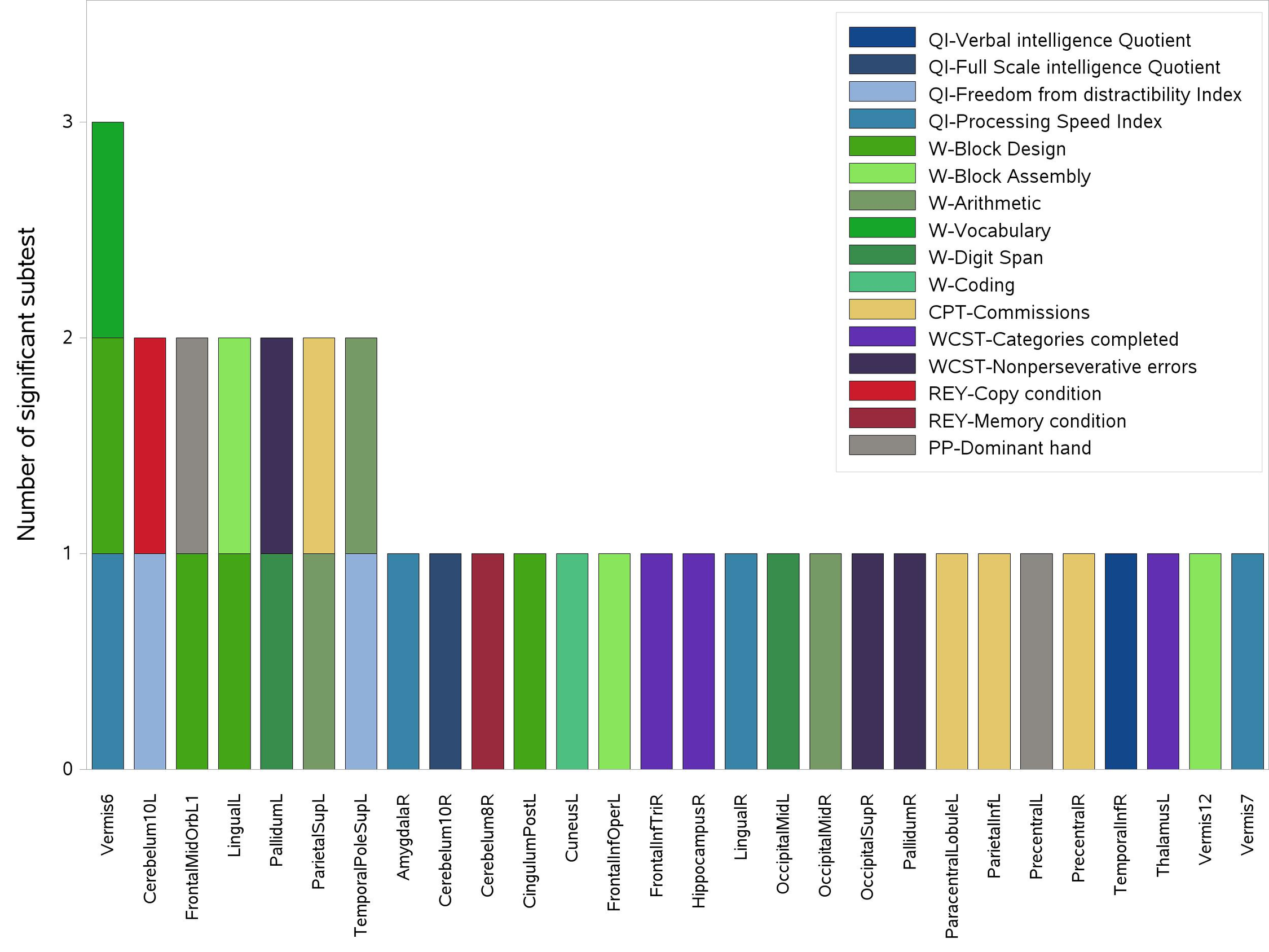

Supplement: S2 Fig — The associations were obtained through the Kruskal Wallis analysis, involving 28 ROIs and 16 different tests. For each ROI reported on the x-axis, all scores showing a significant association with FA are reported on the y-axis in the form of colored boxes. (TIF) [file pone.0247748.s002.tif]

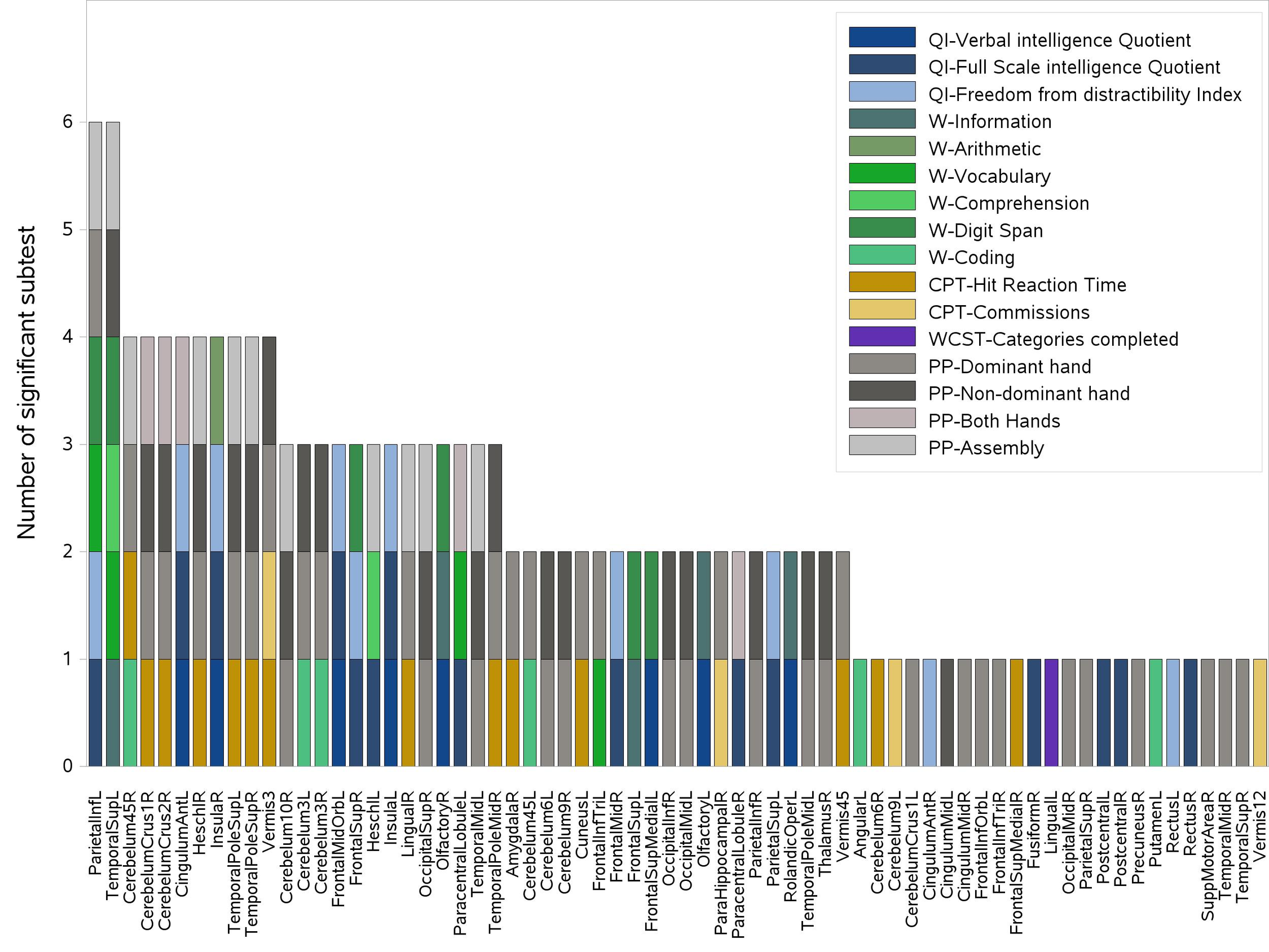

Supplement: S3 Fig — The associations were obtained through the Kruskal Wallis analysis, involving 68 ROIs and 16 different tests. For each ROI reported on the x-axis, all scores showing a significant association with AD are reported on the y-axis in the form of colored boxes. (TIF) [file pone.0247748.s003.tif]

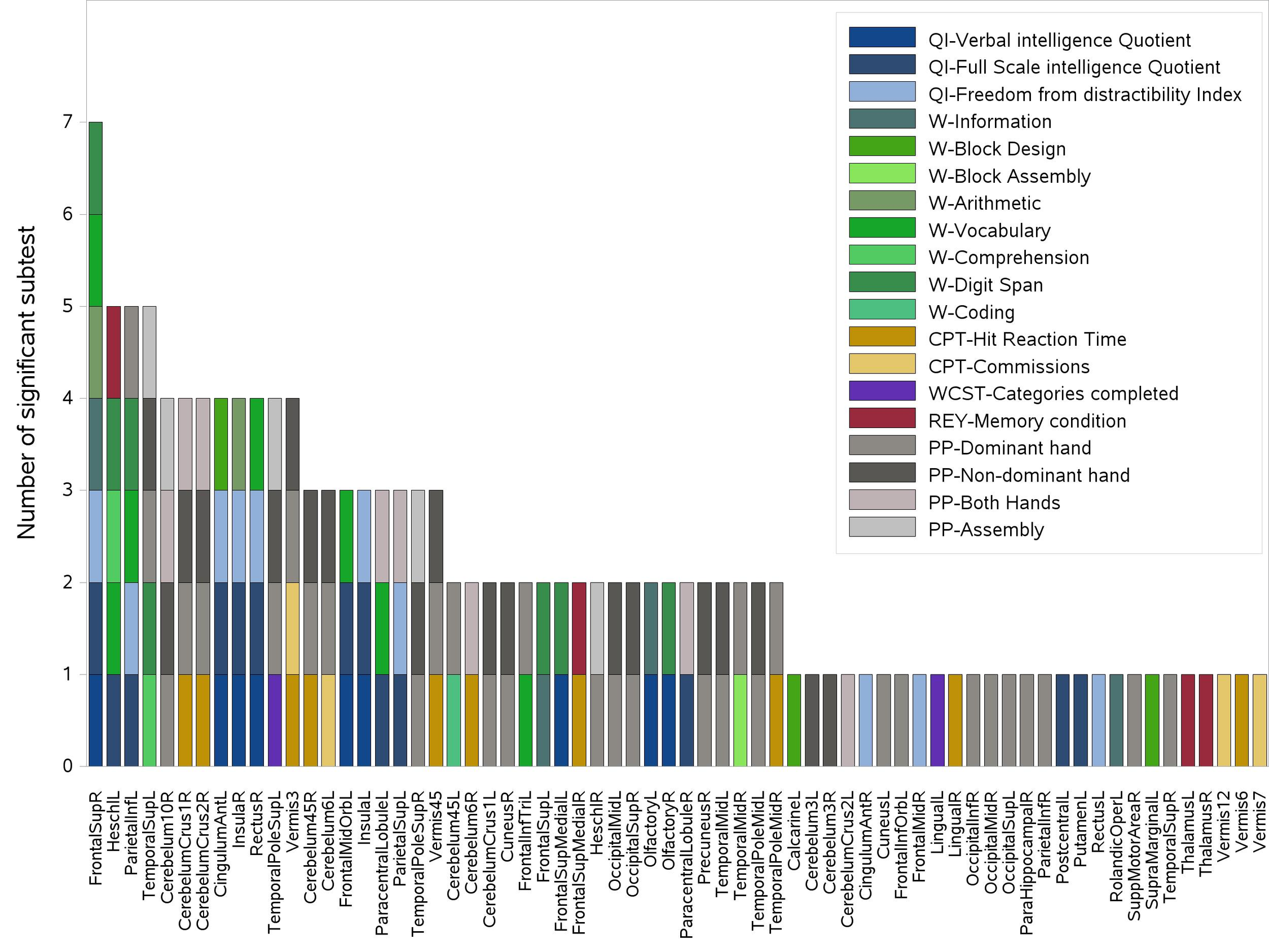

Supplement: S4 Fig — The associations were obtained through the Kruskal Wallis analysis, involving 66 ROIs and 19 different tests. For each ROI reported on the x-axis, all scores showing a significant association with RD are reported on the y-axis in the form of colored boxes. (TIF) [file pone.0247748.s004.tif]

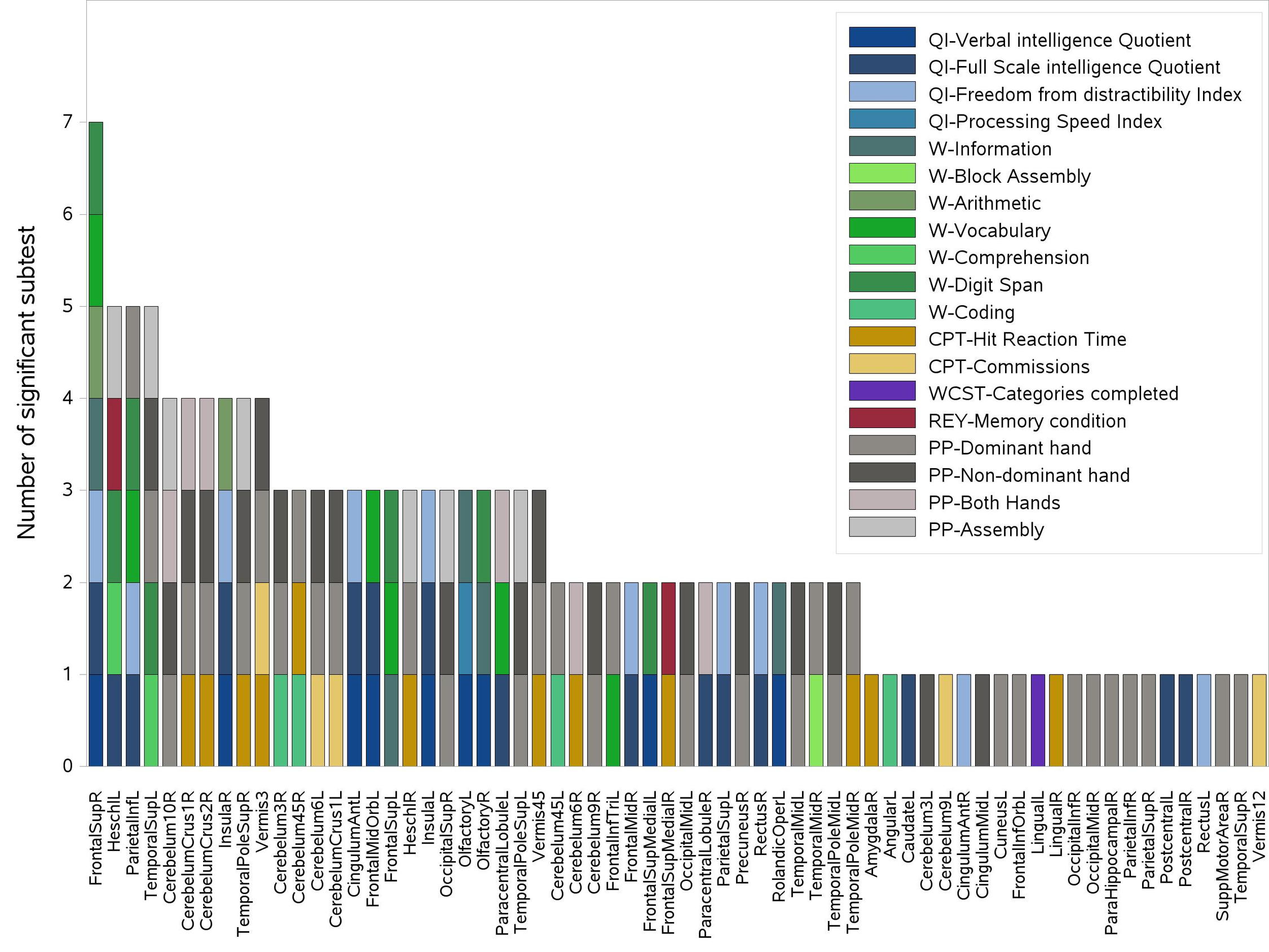

Supplement: S5 Fig — The associations were obtained through the Kruskal Wallis analysis, involving 64 ROIs and 19 different tests. For each ROI reported on the x-axis, all scores showing a significant association with MD are reported on the y-axis in the form of colored boxes. (TIF) [file pone.0247748.s005.tif]

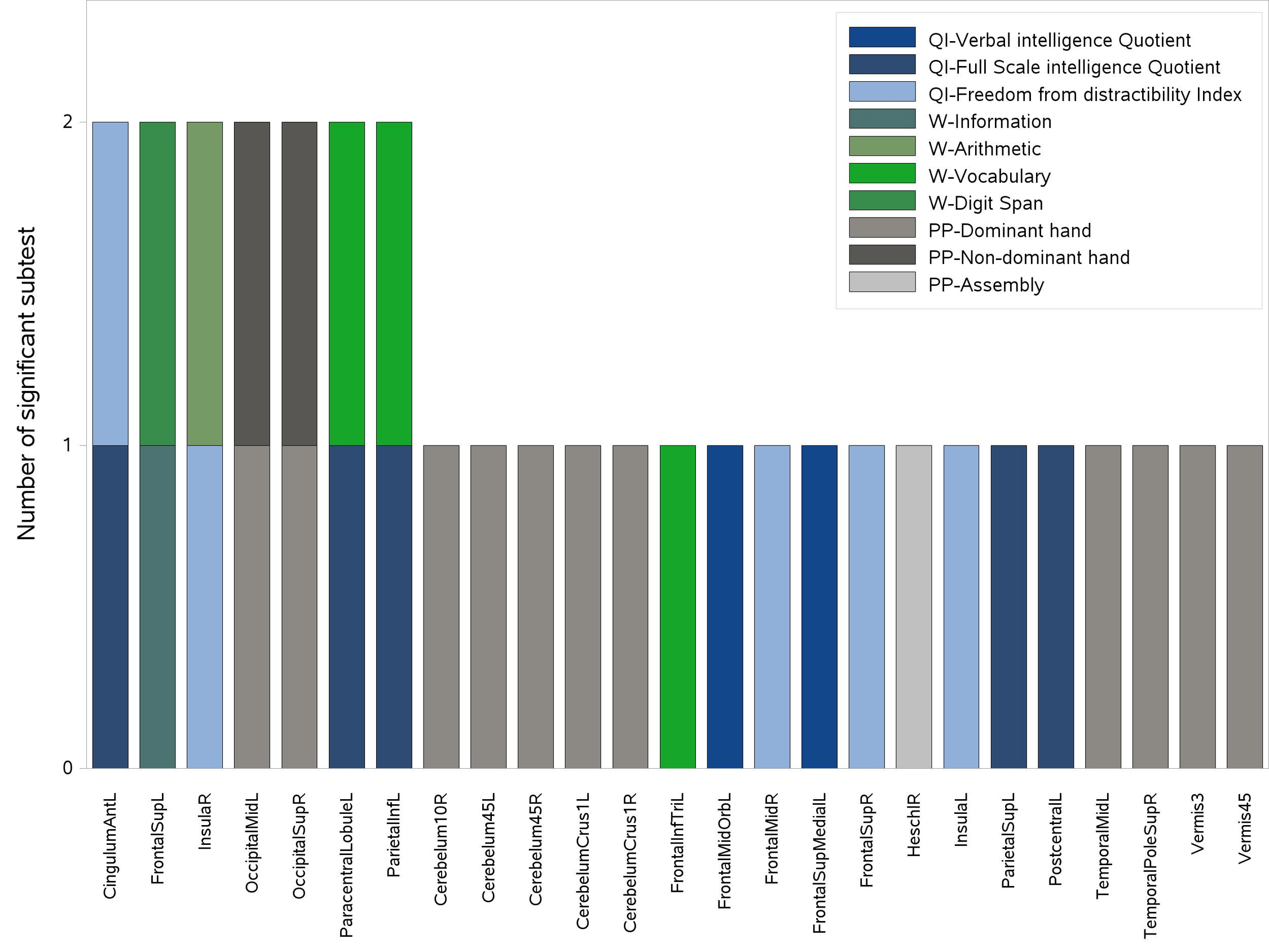

Supplement: S6 Fig — The associations involve 25 ROIs and 10 different tests. For each ROI reported on the x-axis, all scores showing a significant association with dose, MD, RD and AD, are reported on the y-axis in the form of colored boxes. (TIF) [file pone.0247748.s006.tif]

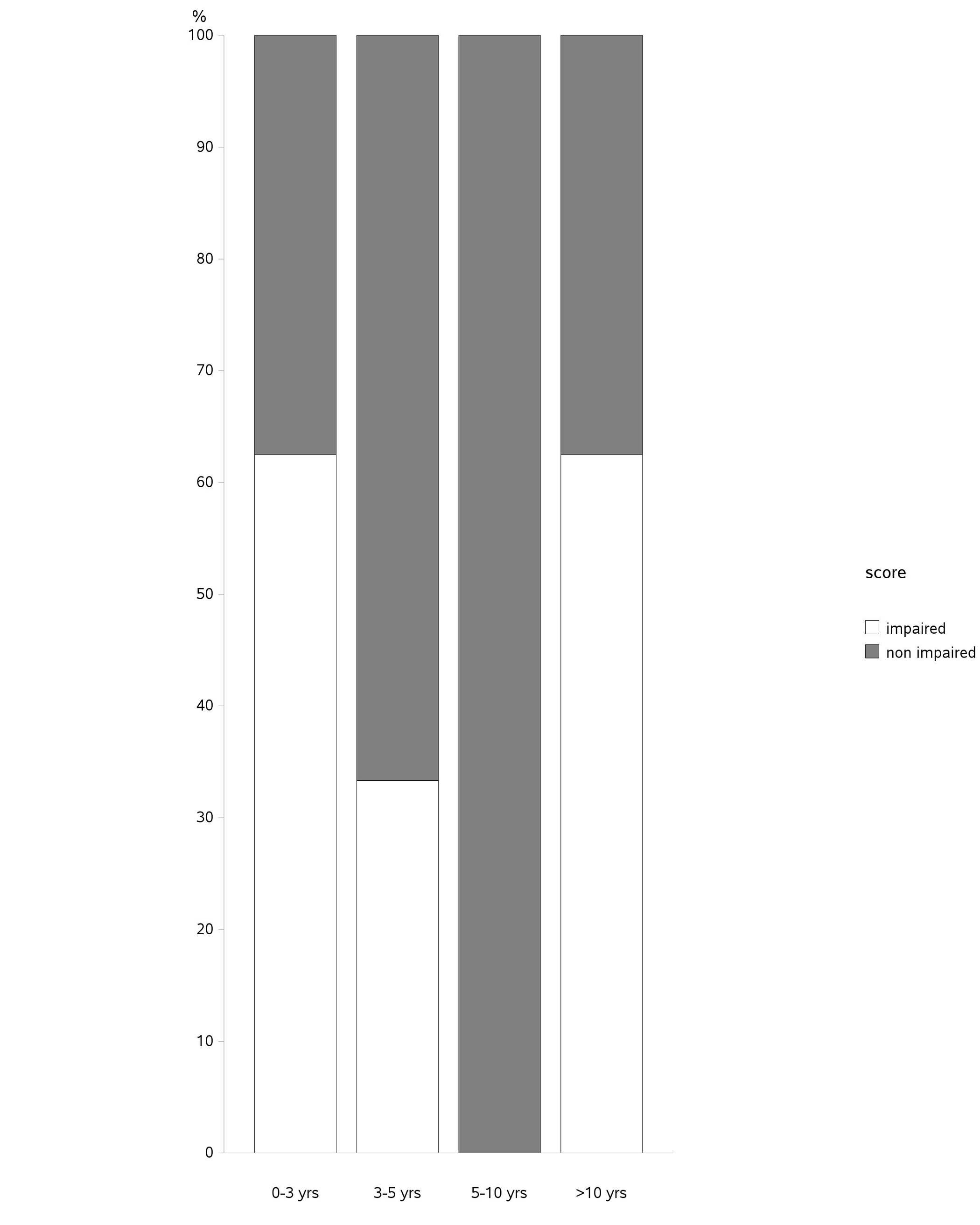

Supplement: S7 Fig — (TIF) [file pone.0247748.s007.tif]

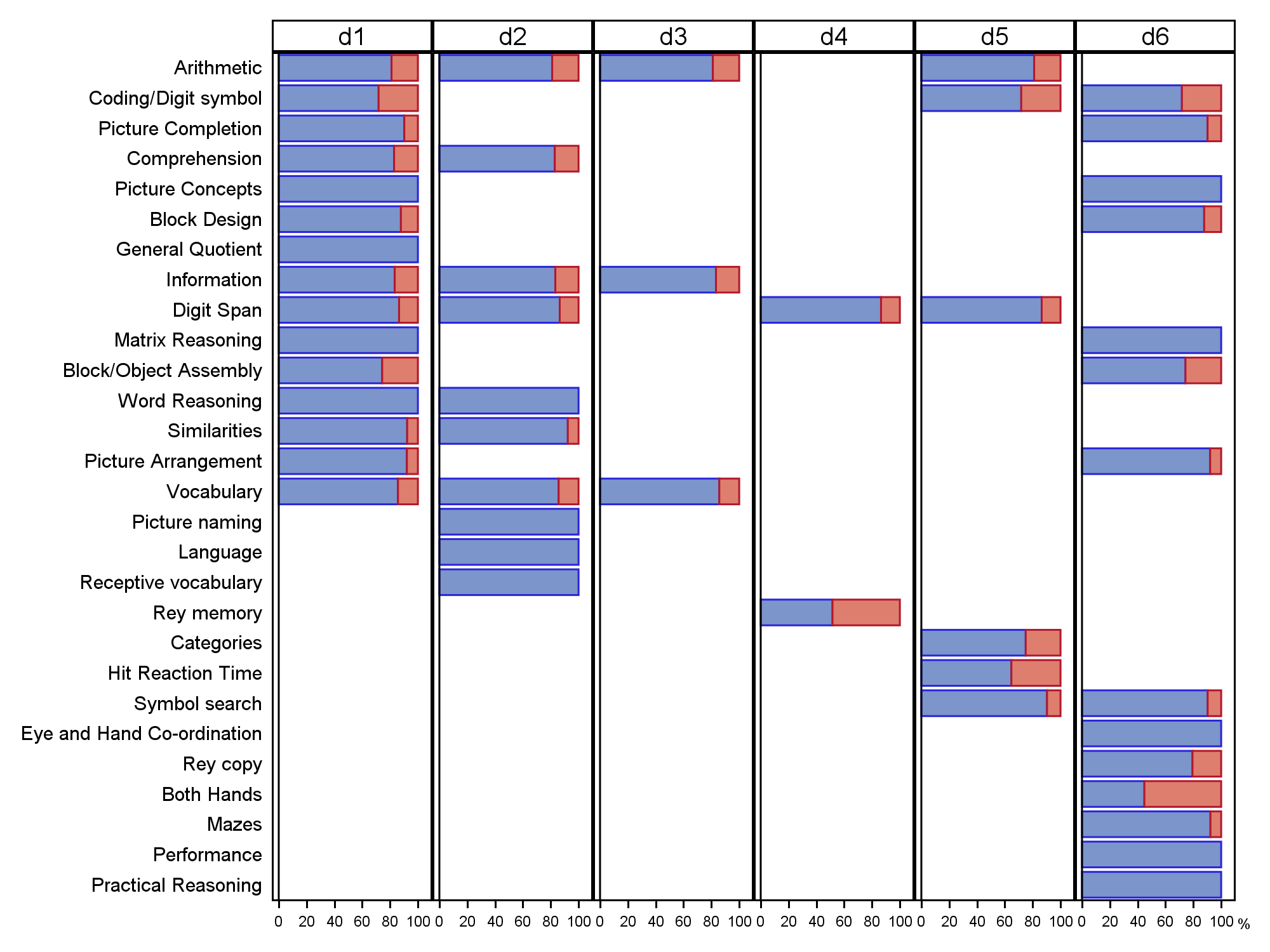

Supplement: S8 Fig — d1: General intellectual abilities, d2: Verbal abilities, d3: School-related abilities, d4: Memory, d5: Executive Functions, d6: Visuo-spatial and visuo-motor abilities. (TIF) [file pone.0247748.s008.tif]
