# Supplementary material for: Retrospective study of late radiation-induced damages after focal radiotherapy for childhood brain tumors
Source: PLoS One. 2021 Feb 26;16(2):e0247748. doi: 10.1371/journal.pone.0247748 (PMC7909688; doi:10.1371/journal.pone.0247748)
Supplement: S1 Table — (PDF) [file pone.0247748.s009.pdf]

		Test/Substest/Indexes					
**Domains**	**Functions**	**Griffiths 0-2 years**	**Griffiths 2-8 years**	**WPPSI III 2,6-3,11 years**	**WPPSI III 4-7,3 years**	**WISC III**	**WAIS R**
General intellectual abilities		QIT	QIT	QIT= QIV +QIP	QIT=QIV +QIP	QIT=QIV +QIP	QIT=QIV +QIP
Verbal abilities	Receptive language	Hearing and language	Language	- Receptive vocabulary<br>- information	- Information<br>- Word reasoning<br>- Comprehension<br>- Receptive vocabulary	- Information<br>- Comprehension<br>- Digit Span	- Information<br>- Digit Span<br>- Arithmetic<br>- Comprehension
	Expressive language			- Picture naming	- Vocabulary<br>- Similarities<br>- Picture naming	- Similarities<br>- Vocabulary	- Similarities<br>- Vocabulary
School-related abilities		/	/	/	- Information<br>- Vocabulary<br>(dai 6 anni)	- Arithmetic<br>- Information<br>- Vocabulary	- Arithmetic<br>- Information<br>- Vocabulary
Memory	Long-term memory	/	/	/	- Rey memory	- Rey memory	- Rey memory
	Short-term memory	/	/	/	- Digit span	- Digit span	- Digit span
Executive Functions	Processing Speed	/	/	/	- Coding<br>- Symbol search	- Coding<br>- Symbol search	- Coding<br>- Symbol search
	Attention				- Hit Reaction Time (HRT) from CPT	- Hit Reaction Time (HRT) from CPT	- Hit Reaction Time (HRT) from CPT
	Working memory	/	/	/	/	- Arithmetic<br>- Digit Span	- Arithmetic<br>- Digit Span
	Flexibility				- Cathegories from WCST	- Cathegories from WCST	- Cathegories from WCST
Visuo-spatial and visuo-motor abilities	Visuo-spatial abilities	- Performance	- Performance<br>- Practical reasoning	/	- Matrix reasoning<br>- Pictures concepts<br>- Picture completion	- Picture completion<br>- Symbol search	- Picture completion
	Visuo-motor abilities	- Locomotor<br>- Eye and hand Coordination	- Locomotor<br>- Eye and hand Coordination	- Block design<br>- Block assembly	- Both hands score from Purdue Pegboard<br>- Rey copy<br>- Block design<br>- Block assembly	- Both hands score from Purdue Pegboard<br>- Rey copy<br>- Coding<br>- Block design<br>- Mazes<br>- Picture arrangement<br>- Object assembly	- Both hands score from Purdue Pegboard<br>- Rey copy<br>- Block design<br>- Object assembly<br>- Digit symbol<br>- Picture arrangement
