# Supplementary material for: Retrospective study of late radiation-induced damages after focal radiotherapy for childhood brain tumors
Source: PLoS One. 2021 Feb 26;16(2):e0247748. doi: 10.1371/journal.pone.0247748 (PMC7909688; doi:10.1371/journal.pone.0247748)
Supplement: S2 Table — (PDF) [file pone.0247748.s010.pdf]

Test	Reference
**Wechsler Preschool and Primary Scale of Intelligence (WPPSI III)**	Wechsler, D. (1998). WPPSI III, Wechsler Preschool and Primary Scale of Intelligence. Third edition. Firenze. Giunti OS.
**Wechsler Intelligence Scale for Children (WISC III)**	Wechsler, D. (2006). WISC III, Wechsler Intelligence Scale for Children. Third edition. Firenze. Giunti Psychometrics.
**Wechsler Adult Intelligence Scale (WAIS-R)**	Wechsler D. (1997). WAIS-R. Wechsler Adult Intelligence Scale Revisited. Firenze. Giunti OS.
**Griffiths Mental Development Scales (GMDS)**	Griffiths R. (2007) Griffiths Mental Development Scales, Extended Revised, 0-2, 2-8 years : manual. Firenze. Giunti OS.
**Rey Complex Figure**	Rey A. (1967). Reattivo della figura complessa: Manuale. Firenze. Giunti OS.
**Conners Kiddie Continuous Performance Test (K-CPT)**	Conners K.C. (2001, 2006). Conners' Kiddie Continuous Performance Test (K-CPT 2). Technical Guide and software Manual. MHS.
**Continuous Performance Test (CPT)**	Conners K.C. (2002). Conners' Continuous Performance Test (CPT II). Technical Guide and software Manual. MHS.
**Modified Card Sorting Test (MCST)**	Heaton R., Gordon J., ., Chelune G., Talley J., Kay G., Curtiss G. (2003). MCST. Modified card Sorting Test. Manuale. Firenze. Giunti OS.
**Wisconsin Card Sorting Test (WCST)**	Heaton R., Chelune G., Talley J., Kay G., Curtiss G. (2000). WCST. Wisconsin card Sorting Test. Forma complete revisionata: Manuale. Firenze. Giunti OS.
**Perdue Pegboard (PP)**	Purdue Pegboard Test. User Instructions. (2015). Lafayette Instrument. Free download: http://lafayetteevaluation.com/products/purdue-pegboard
