# Supplementary material for: Retrospective study of late radiation-induced damages after focal radiotherapy for childhood brain tumors
Source: PLoS One. 2021 Feb 26;16(2):e0247748. doi: 10.1371/journal.pone.0247748 (PMC7909688; doi:10.1371/journal.pone.0247748)
Supplement: S3 Table — Note: x: impaired score; -: not applicable for patient age or not administered; FDI: Freedom from Distractability Index; FSIQ: Full Scale Intelligent Quotient; GMDS: Griffiths Mental Development Scales; K/CPT: Kiddie and Continuous Performance Test; M/WCST: Modified and Wisconsin Card Sorting Test; PIQ: Performance IQ; PP: Purdeue Pegboard; PSI: Processing Speed Index; REY: Rey Complex Figure; VIQ: Verbal IQ; W/GMDS: Wechsler and Griffiths Mental Development Scales; WISC: Wechsler Intelligence Scale for Children 3rd Edition; WPPSI: Wechsler Preschool and Primary cale of Intelligence, 3rd Edition. (PDF) [file pone.0247748.s011.pdf]

		Baseline Evaluations																		
		Patients																		
Test	Subtest/Indexes	1	2	4	6	7	8	9	10	11	14	17	19	22	24	26	27	32	38	TOT
W/GMDS TIQ	FSIQ													x		x				**2**
Wechsler Indexes	VIQ			-						x		-	-	-		-				**1**
	PIQ			-								-	-	-		-				**0**
	PSI	-	-	-	-	-	-	-	-			-	-	-	-	-			x	**1**
	FDI	-	-	-	-	-	-	-	-			-	-	-	-	-	-			**0**
GMDS	Locomotor	-	-	x	-	-	-	-	-	-	-	x	-	x	-	x	-	-	-	**4**
	Social Behaviour	-	-		-	-	-	-	-	-	-			x	-		-	-	-	**1**
	Language	-	-		-	-	-	-	-	-	-			x	-		-	-	-	**1**
	EH Coordination	-	-		-	-	-	-	-	-	-			x	-	x	-	-	-	**2**
	Performance	-	-		-	-	-	-	-	-	-			x	-		-	-	-	**1**
	Practical Reasoning	-	-		-	-	-	-	-	-	-			-	-		-	-	-	**0**
Wechsler Common Subtests	Block Design			-								-	-	-		-				**0**
	Information			-								-	-	-		-				**0**
	Vocabulary			-						x		-	-	-		-				**1**
	Symbol Search			-								-	-	-		-				**0**
	Coding	x		-								-	-	-		-				**1**
	Comprehension			-								-	-	-		-				**0**
	Similarities			-						x	x	-	-	-		-				**2**
	Picture Completion			-								-	-	-		-				**0**
	Object Assembly			-								-	-	-		-				**0**
WPPSI	Matrix Reasoning	-		-			-	-		-	-	-	-	-		-		-	-	**0**
	Pictures Concepts	-		-			-	-		-	-	-	-	-		-		-	-	**0**
	Word Reasononig	-		-			-	-		-	-	-	-	-		-		-	-	**0**
	ReceptiveVocabulary	-		-			-	-		-	-	-	-	-		-		-	-	**0**
	Picture Naming	-		-			-	-		-	-	-	-	-		-		-	-	**0**
WISC	Arithmetic		-	-	-	-			-			-	-	-	-	-	-			**0**
	Digit Span		-	-	-	-			-			-	-	-	-	-	-			**0**
	Picture Arrangement		-	-	-	-	x		-		x	-	-	-	-	-	-			**2**
	Mazes		-	-	-	-			-		x	-	-	-	-	-	-			**1**
K/CPT	HRT			-	-	x		x				-	-	-	x	-	-			**3**
	Omissions			-	-	x						-	-	-	x	-	-			**2**
	Commissions	x		-	-							-	-	-		-	-			**1**
M/WCST	Category	-	-	-	-	-					x	-	-	-		-	-			**1**
	Total Errors	-	-	-	-	-						-	-	-		-	-			**0**
	Pers. Responses	-	-	-	-	-		x				-	-	-		-	-			**1**
REY	Copy	-	-	-	-	-				x	x	-	-	-	-	-	-	-	-	**2**
	Memory	-	-	-	-	-	x			x	x	-	-	-	-	-	-	-	-	**3**
PP	Dominant Hand	-	-	-	-	-	-	-	-	x	x	-	-	-	-	-	-	x	x	**4**
	Non-Dominant Hand	-	-	-	-	-	-	-	-	x	x	-	-	-	-	-	-	x	x	**4**
	Both Hands	-	-	-	-	-	-	-	-	x	x	-	-	-	-	-	-	x	x	**4**
	Assembly	-	-	-	-	-	-	-	-	x	x	-	-	-	-	-	-	x	x	**4**
	**TOT**	**2**	**0**	**1**	**0**	**2**	**2**	**2**	**0**	**9**	**10**	**1**	**0**	**6**	**2**	**3**	**0**	**4**	**5**	
