# Supplementary material for: Retrospective study of late radiation-induced damages after focal radiotherapy for childhood brain tumors
Source: PLoS One. 2021 Feb 26;16(2):e0247748. doi: 10.1371/journal.pone.0247748 (PMC7909688; doi:10.1371/journal.pone.0247748)
Supplement: S4 Table — (PDF) [file pone.0247748.s012.pdf]

MRI sequences	
Scanner	Philips® (Best, The Netherlands) model Achieva, 3T, 32-channel head coil
T1-weighted	TR=8.2 ms, TE=3.8 ms, flip angle=8°, FOV=210x210x170 mm^3, acquired voxel size=1x1x1 mm^3
T2-weighted	Fat-suppressed TSE sequence: TR=3000 ms, TE=100 ms, FOV=224x224 mm^2, acquired matrix=112x112, slice thickness=1.7 mm, voxel size=2x2x1.7 mm^3)
DTI	Multi-shell 2D T2-weighted EPI sequence including 15 directions at b=300 s/mm2, 53 directions at b=1100 s/mm^2 and 8 volumes at b=0 s/mm^2.<br>TR=8645 ms, TE=63 ms, flip angle=90°, FOV=224x224 mm^2, acquired matrix=112x112, slice thickness=2 mm, final voxel size=2x2x2 mm^3.
