# Supplementary material for: Retrospective study of late radiation-induced damages after focal radiotherapy for childhood brain tumors
Source: PLoS One. 2021 Feb 26;16(2):e0247748. doi: 10.1371/journal.pone.0247748 (PMC7909688; doi:10.1371/journal.pone.0247748)
Supplement: S5 Table — Abbreviations: ROI, regions of interest. From Tzourio-Mazoyer et al. [39]. (PDF) [file pone.0247748.s013.pdf]

ROI Acronym	Anatomical description	ROI Acronym	Anatomical description
**Amygdala**	Sub cortical gray nuclei, amygdala	**Occipital_Inf**	Occipital lobe lateral surface, inferior occipital gyrus
**Angular**	Parietal lobe lateral surface, angular gyrus	**Occipital_Mid**	Occipital lobe lateral surface, middle occipital gyrus
**Calcarine**	Occipital lobe medial and inferior surfaces, calcarine fissure and surrounding cortex	**Occipital_Sup**	Occipital lobe lateral surface, superior occipital gyrus
**Caudate**	Sub cortical gray nuclei, caudate nucleus	**Olfactory**	Frontal lobe orbital surface, olfactory cortex
**Cerebelum_10**	Lobule X of cerebellum	**Pallidum**	Sub cortical gray nuclei, lenticular nucleus, pallidum
**Cerebelum_3**	Lobule III of cerebellum	**Paracentral_Lobule**	Frontal lobe medial surface, paracentral lobule PCL
**Cerebelum_4_5**	Lobule IV and V of cerebellum	**ParaHippocampal**	Limbic lobe, parahippocampal gyrus
**Cerebelum_6**	Lobule VI of cerebellum	**Parietal_Inf**	Parietal lobe lateral surface, inferior parietal, but supramarginal and angular gyri
**Cerebelum_7b**	Lobule VIIB of cerebellum	**Parietal_Sup**	Parietal lobe lateral surface, superior parietal gyrus
**Cerebelum_8**	Lobule VIII of cerebellum	**Postcentral**	Central region, postcentral gyrus
**Cerebelum_9**	Lobule IX of cerebellum	**Precentral**	central region precentral gyrus
**Cerebelum_Crus1**	Crus I of cerebellum	**Precuneus**	Parietal lobe medial surface, precuneus
**Cerebelum_Crus2**	Crus II of cerebellum	**Putamen**	Sub cortical gray nuclei, lenticular nucleus, putamen
**Cingulum_Ant**	Limbic lobe, anterior cingulate and paracingulate gyri	**Rectus**	Frontal lobe orbital surface, gyrus rectus
**Cingulum_Mid**	Limbic lobe, median cingulate and paracingulate gyri	**Rolandic_Oper**	Central region, rolandic operculum
**Cingulum_Post**	Limbic lobe, posterior cingulate gyrus	**Supp_Motor_Area**	Frontal lobe medial surface, supplementary motor area SMA
**Cuneus**	Occipital lobe medial and inferior surfaces,cuneus	**SupraMarginal**	Parietal lobe lateral surface, supramarginal gyrus
**Frontal_Inf_Oper**	Frontal lobe lateral surface, inferior frontal gyrus, opercular part	**Temporal_Inf**	Temporal lobe lateral surface, inferior temporal gyrus
**Frontal_Inf_Orb**	Frontal lobe orbital surface, inferior frontal gyrus, orbital part	**Temporal_Mid**	Temporal lobe lateral surface, middle temporal gyrus
**Frontal_Inf_Tri**	Frontal lobe lateral surface, inferior frontal gyrus, triangular part	**Temporal_Pole_Mid**	Limbic lobe, temporal pole: middle temporal gyrus
**Frontal_Mid**	Frontal lobe lateral surface, middle frontal gyrus	**Temporal_Pole_Sup**	Limbic lobe, temporal pole: superior temporal gyrus
**Frontal_Mid_Orb**	Frontal lobe orbital surface, middle frontal gyrus, orbital part	**Temporal_Sup**	Temporal lobe lateral surface, superior temporal gyrus
**Frontal_Mid_Orb**	Frontal lobe orbital surface, superior frontal gyrus, medial orbital	**Thalamus**	Sub cortical gray nuclei, thalamus
**Frontal_Sup**	Frontal lobe lateral surface, superior frontal gyrus, dorsolateral	**Vermis_1_2**	Lobule I and II of vermis
**Frontal_Sup_Medial**	Frontal lobe medial surface, superior frontal gyrus, medial	**Vermis_10**	Lobule X of vermis
**Frontal_Sup_Orb**	Frontal lobe orbital surface, superior frontal gyrus, orbital part	**Vermis_3**	Lobule III of vermis
**Fusiform**	Occipital lobe medial and inferior surfaces, fusiform gyrus	**Vermis_4_5**	Lobule IV and V of vermis
**Heschl**	Temporal lobe lateral surface, heschl gyrus	**Vermis_6**	Lobule VI of vermis
**Hippocampus**	Limbic lobe, hippocampus	**Vermis_7**	Lobule VII of vermis
**Insula**	Insula	**Vermis_8**	Lobule VIII of vermis
**Lingual**	Occipital lobe medial and inferior surfaces, lingual gyrus	**Vermis_9**	Lobule IX of vermis
