# Supplementary material for: Retrospective study of late radiation-induced damages after focal radiotherapy for childhood brain tumors
Source: PLoS One. 2021 Feb 26;16(2):e0247748. doi: 10.1371/journal.pone.0247748 (PMC7909688; doi:10.1371/journal.pone.0247748)
Supplement: S6 Table — (PDF) [file pone.0247748.s014.pdf]

ROI_name	excluded number	ROI_name	excluded number	ROI_name	excluded number
Vermis_8	27/44	Cingulum_Mid_L	8/44	Frontal_Inf_Tri_L	3/44
Cerebelum_9_L	24/44	Heschl_L	8/44	Frontal_Inf_Tri_R	3/44
Cerebelum_9_R	23/44	Rectus_L	8/44	Frontal_Mid_L	3/44
Vermis_1_2	21/44	Amygdala_R	7/44	Frontal_Mid_Orb_L	3/44
Cerebelum_3_L	20/44	Cingulum_Ant_L	7/44	Frontal_Sup_Medial_L	3/44
Cerebelum_3_R	19/44	Cingulum_Ant_R	7/44	Frontal_Sup_Medial_R	3/44
Vermis_3	19/44	Cingulum_Mid_R	7/44	Frontal_Sup_Orb_L	3/44
Vermis_4_5	19/44	Fusiform_R	7/44	Parietal_Inf_R	3/44
Thalamus_L	18/44	Heschl_R	7/44	Parietal_Sup_L	3/44
Pallidum_L	17/44	Lingual_R	7/44	Precentral_R	3/44
Pallidum_R	17/44	Calcarine_L	6/44	Precuneus_L	3/44
Cerebelum_10_L	16/44	Calcarine_R	6/44	Precuneus_R	3/44
Cerebelum_10_R	16/44	Fusiform_L	6/44	Rolandic_Oper_L	3/44
Cerebelum_4_5_L	16/44	Rectus_R	6/44	Temporal_Pole_Sup_R	3/44
Hippocampus_L	16/44	Frontal_Mid_Orb_R_1	5/44	Temporal_Sup_L	3/44
Vermis_7	16/44	Lingual_L	5/44	Cerebelum_Crus1_L	3/44
Caudate_L	15/44	Olfactory_L	5/44	Cuneus_R	2/44
Hippocampus_R	15/44	Olfactory_R	5/44	Frontal_Inf_Oper_L	2/44
Caudate_R	14/44	Rolandic_Oper_R	5/44	Frontal_Inf_Orb_L	2/44
Cerebelum_4_5_R	14/44	Supp_Motor_Area_L	5/44	Frontal_Mid_Orb_R	2/44
Cerebelum_8_R	14/44	Angular_R	5/44	Frontal_Sup_L	2/44
Cingulum_Post_R	14/44	Frontal_Inf_Orb_R	4/44	Occipital_Mid_R	2/44
ParaHippocampal_R	14/44	Frontal_Mid_Orb_L_1	4/44	Occipital_Sup_R	2/44
Putamen_R	14/44	Frontal_Mid_R	4/44	Parietal_Inf_L	2/44
Cerebelum_8_L	13/44	Frontal_Sup_Orb_R	4/44	Parietal_Sup_R	2/44
Cingulum_Post_L	13/44	Frontal_Sup_R	4/44	Postcentral_R	2/44
ParaHippocampal_L	13/44	Occipital_Inf_R	4/44	SupraMarginal_L	2/44
Putamen_L	13/44	Paracentral_Lobule_L	4/44	SupraMarginal_R	2/44
Vermis_6	13/44	Postcentral_L	4/44	Temporal_Inf_L	2/44
Cerebelum_7b_R	12/44	Precentral_L	4/44	Temporal_Mid_L	2/44
Thalamus_R	12/44	Supp_Motor_Area_R	4/44	Temporal_Mid_R	2/44
Paracentral_Lobule_R	10/44	Temporal_Inf_R	4/44	Temporal_Pole_Mid_L	2/44
Cerebelum_6_L	9/44	Angular_L	4/44	Temporal_Pole_Mid_R	2/44
Cerebelum_6_R	9/44	Cerebelum_Crus1_R	3/44	Temporal_Pole_Sup_L	2/44
Insula_L	9/44	Cerebelum_Crus2_L	3/44	Occipital_Inf_L	2/44
Insula_R	9/44	Cerebelum_Crus2_R	3/44	Occipital_Mid_L	1/44
Amygdala_L	8/44	Cuneus_L	3/44	Occipital_Sup_L	1/44
Cerebelum_7b_L	8/44	Frontal_Inf_Oper_R	3/44	Temporal_Sup_R	1/44
