# Supplementary material for: Retrospective study of late radiation-induced damages after focal radiotherapy for childhood brain tumors
Source: PLoS One. 2021 Feb 26;16(2):e0247748. doi: 10.1371/journal.pone.0247748 (PMC7909688; doi:10.1371/journal.pone.0247748)
Supplement: S7 Table — (PDF) [file pone.0247748.s015.pdf]

subtest & indexes	Frequency of score impaired	Frequency of score non impaired
Arithmetic	7	30
Assembly	8	22
Coding	11	28
Comprehension	7	34
Copy	7	27
Categories	7	21
Block design	5	36
Both-hands	16	13
Non-perseverative Errors	5	14
Reaction Times	6	11
Freedom from distractability	6	21
Information	7	35
Processing speed	8	23
Recall	15	16
Digit span	5	32
Dominant hand	11	19
Non Dominant hand	12	18
Object assembly	10	29
Vocabulary	6	36
Commissions	7	16
TIQ	5	38
VIQ	8	35
