# Supplementary material for: Retrospective study of late radiation-induced damages after focal radiotherapy for childhood brain tumors
Source: PLoS One. 2021 Feb 26;16(2):e0247748. doi: 10.1371/journal.pone.0247748 (PMC7909688; doi:10.1371/journal.pone.0247748)
Supplement: S1 File — (PDF) [file pone.0247748.s016.pdf]

S1 File. Overview of the baseline neurocognitive evaluation results.

Baseline results were available for 18 patients on 45 before the beginning of the radiation therapy, but after the neurosurgery (See S3 Table). Patients were assessed with a different number of test, according to their age. Since on some occasions, a few tests were not administered because of time limitations or patient illness, percentages were calculated on the number of actually undergone tests for each patient.

Intellectual functioning was assessed for 5 children with the Griffith Mental Development Scales (GMDS), for 6 patients with the Wechsler Preschool and Primary Scale for Children, 3rd Edition (WPPSI-III) and for 7 subjects with the Wechsler Intelligence Scale for Children, 3rd Edition (WISC-III). Only 2 patients (11,11%) had an impaired total IQ (FSIQ) before radiotherapy.

Eleven subjects received attention evaluation with the Kiddie or Continuous Performance Test; 8 patients underwent the Modified or Wisconsin Card Sorting Test to evaluate executive functions; the Complex Rey Figure was used to assess memory and praxic abilities in 5 patients and finally 4 patients completed the Purdue Pegboard to examine their praxic and fine-motor abilities.

The most frequent impaired scores were the Locomotor scale from the GMDS (4/4 patients, 100,00%), the Hit Reaction Time from CPT (3/11; 27,00%) the Memory condition of the Rey Complex Figure (3/5 patients; 60%) and the Purdue Pegboard (all 4 scores, 4/4 patients; 100%).

From a qualitative single patient analysis, 4 subjects had more than the 26% of the evaluation falling below the average.

Patient 14 had 10 scores falling below the average (33,33%). The most impaired domains were executive functions, memory and fine-motor dexterity, while general intellectual functioning was preserved.

Patients 11 also showed several scores (i.e., 9/34, 26,47%) falling below the norm and more specifically memory and praxic abilities were the functions more impaired, together with verbal functioning.

Patient 22 had 6 impaired scores (on 6; 100%) and Patients 26 had 3 impaired scores (on 7, 42,84%)

The remaining sample had less than 26% impaired scores and of these 14 patients 5 had 100% of the evaluation unimpaired.
